# An Unsupervised Machine Learning Approach to Evaluating the Association of Symptom Clusters With Adverse Outcomes Among Older Adults With Advanced Cancer: A Secondary Analysis of a Randomized Clinical Trial

**DOI:** 10.1001/jamanetworkopen.2023.4198

**Published:** 2023-03-22

**Authors:** Huiwen Xu, Mostafa Mohamed, Marie Flannery, Luke Peppone, Erika Ramsdale, Kah Poh Loh, Megan Wells, Leah Jamieson, Victor G. Vogel, Bianca Alexandra Hall, Karen Mustian, Supriya Mohile, Eva Culakova

**Affiliations:** 1 School of Public and Population Health, University of Texas Medical Branch, Galveston; 2 Sealy Center on Aging, University of Texas Medical Branch, Galveston; 3 Department of Public Health Sciences, University of Rochester Medical Center, Rochester, New York; 4 James P. Wilmot Cancer Institute, Division of Hematology/Oncology, Department of Medicine, University of Rochester Medical Center, Rochester, New York; 5 School of Nursing, University of Rochester Medical Center, Rochester, New York; 6 Department of Surgery, Supportive Care in Cancer, University of Rochester Medical Center, Rochester, New York; 7 Metro Minnesota Community Oncology Research Program, St Louis Park, Minnesota; 8 Geisinger Cancer Institute National Cancer Institute Community Oncology Research Program, Danville, Pennsylvania

## Abstract

**Importance:**

Older adults with advanced cancer who have high pretreatment symptom severity often experience adverse events during cancer treatments. Unsupervised machine learning may help stratify patients into different risk groups.

**Objective:**

To evaluate whether clusters identified from baseline patient-reported symptom severity were associated with adverse outcomes.

**Design, Setting, and Participants:**

This secondary analysis of the Geriatric Assessment Intervention for Reducing Toxicity in Older Patients With Advanced Cancer (GAP70+) Trial (2014-2019) included patients who completed the National Cancer Institute Patient-Reported Outcomes version of the Common Terminology Criteria for Adverse Events (PRO-CTCAE) before starting a new cancer treatment regimen and received care at community oncology sites across the United States. An unsupervised machine learning algorithm (k-means with Euclidean distance) clustered patients based on similarities of baseline symptom severities. Clustering variables included severity items of 24 PRO-CTCAE symptoms (range, 0-4; corresponding to none, mild, moderate, severe, and very severe). Total severity score was calculated as the sum of 24 items (range, 0-96). Whether the clusters were associated with unplanned hospitalization, death, and toxic effects was then examined. Analyses were conducted in January and February 2022.

**Exposures:**

Symptom severity.

**Main Outcomes and Measures:**

Unplanned hospitalization over 3 months (primary), all-cause mortality over 1 year, and any clinician-rated grade 3 to 5 toxic effect over 3 months.

**Results:**

Of 718 enrolled patients, 706 completed baseline PRO-CTCAE and were included (mean [SD] age, 77.2 [5.5] years, 401 [56.8%] male patients; 51 [7.2%] Black and 619 [87.8%] non-Hispanic White patients; 245 [34.7%] with gastrointestinal cancer; 175 [24.8%] with lung cancer; mean [SD] impaired Geriatric Assessment domains, 4.5 [1.6]). The algorithm classified 310 (43.9%), 295 (41.8%), and 101 (14.3%) into low-, medium-, and high-severity clusters (within-cluster mean [SD] severity scores: low, 6.3 [3.4]; moderate, 16.6 [4.3]; high, 29.8 [7.8]; *P* < .001). Controlling for sociodemographic variables, clinical factors, study group, and practice site, compared with patients in the low-severity cluster, those in the moderate-severity cluster were more likely to experience hospitalization (risk ratio, 1.36; 95% CI, 1.01-1.84; *P* = .046). Moderate- and high-severity clusters were associated with a higher risk of death (moderate: hazard ratio, 1.31; 95% CI, 1.01-1.69; *P* = .04; high: hazard ratio, 2.00; 95% CI, 1.43-2.78; *P* < .001), but not toxic effects.

**Conclusions and Relevance:**

In this study, unsupervised machine learning partitioned patients into distinct symptom severity clusters; patients with higher pretreatment severity were more likely to experience hospitalization and death.

**Trial Registration:**

ClinicalTrials.gov Identifier: NCT02054741

## Key Points

**Question:**

Can unsupervised machine learning identify older adults with advanced cancer who are at high risk of adverse outcomes based on patient-reported symptoms prior to cancer treatments?

**Findings:**

In this secondary analysis of a randomized clinical trial with 706 older adults with advanced cancer, a k-means algorithm identified 3 patient clusters characterized by symptom severity (low, moderate, and high) that were associated with increased risk of unplanned hospitalization and death.

**Meaning:**

These findings suggest that machine learning may be used to guide the development of risk stratification tools with the potential to assist clinicians in identifying older adults with high risk of hospitalization and death.

## Teaser

This secondary analysis of a randomized clinical trial uses unsupervised machine learning to evaluate the association between symptom severity clusters among older patients with advanced cancer and adverse outcomes.

## Introduction

Older adults with advanced cancer usually present with a wide range of physical and psychological symptoms, such as insomnia, fatigue, and pain, prior to cancer treatments.^1,2,3,4,5^ Those patients are more vulnerable to developing symptoms compared with younger adults because of aging-related conditions (eg, disability and/or preexisting comorbidities), frailty, and lower physiological reserve with organ function.^2,6,7,8^ Preexisting symptoms have been found to be associated with adverse outcomes during and after active treatments, including poor treatment tolerability (ie, defined as the degree to which overt adverse effects can be tolerated by the patient^2^), functional deterioration, and worse quality of life.^9,10^ On the other hand, some robust older adults may be undertreated due to potential bias toward age among clinicians. The American Society of Clinical Oncology recommends using the geriatric assessment (GA) to assess vulnerabilities in older adults undergoing chemotherapy.^11^

In recent years, patient-reported outcomes (PROs) have been increasingly used as a measure to capture symptom burden in patients with cancer.^12,13,14^ Symptom monitoring and management using PROs have been shown to improve quality of life, reduce emergency department visits, and maintain cancer treatments.^2,13,15^ Hence, the Food and Drug Administration and Friends of Cancer Research have identified PROs as a key end point related to tolerability assessment for clinical trials.^16^ As a complement to the clinician-rated Common Terminology Criteria for Adverse Events (CTCAE), the National Cancer Institute (NCI) developed the PRO version of the CTCAE (PRO-CTCAE) to seek information from patients.^14^ The NCI PRO-CTCAE item library includes 78 symptom terms assessing up to 4 attributes: presence or absence, frequency, severity, and interference with daily activities.^17^

To standardize the analysis of PRO-CTCAE data, the NCI formed a Cancer Treatment Tolerability Consortium with 4 research teams in 2018, as part of the Cancer Moonshot initiative.^2,18^ PRO-CTCAE items were developed to be analyzed and reported individually^19^; however, one methodologic initiative is examining a summative score that would capture overall symptom burden at baseline or cumulative symptomatic toxic effects during treatment. One potential approach is to use machine learning algorithms^20^ in addition to traditional methods, such as total score (eg, the scoring of MD Anderson Symptom Inventory).^21,22^ Specifically, unsupervised machine learning, a data mining method that aims to detect unknown patterns in data without the need for prior human knowledge and intervention,^20,23,24^ may achieve this purpose. Researchers have become increasingly interested in applying unsupervised machine learning to identify cancer symptom subgroups.^25,26,27^ To our knowledge, no study has applied those algorithms to PRO-CTCAE data among older adults with advanced cancer.

To mitigate knowledge gaps, this study aimed to (1) identify patient clusters based on pretreatment PRO-CTCAE severity items using an unsupervised machine learning approach; (2) examine differences in patient characteristics and individual and total symptom severity by clusters; and (3) evaluate the longitudinal associations of patient clusters with unplanned hospitalization, overall mortality, and clinician-rated toxic effects. We hypothesized that the patterns of unplanned hospitalizations, mortality, and toxic effects will differ by symptom clusters.

## Methods

### Data Source

This secondary analysis used data from a nationwide, multicenter, cluster-randomized study that found that providing GA information to community oncologists reduced clinician-rated grade 3 to 5 toxic effects in older adults with advanced cancer starting a new cancer treatment regimen (GAP70+ study).^8^ The trial protocol appears in Supplement 1. Patients were recruited through the University of Rochester National Cancer Institute Community Oncology Research Program (URCC NCORP) Research Base. In total, the trial enrolled 718 patients between July 2014 and March 2019. All patients provided written informed consent. Our analysis included 706 participants who completed the PRO-CTCAE at baseline. Patients from both the intervention and control groups were included in the analysis.^8^ The University of Rochester and all participating practices obtained approval from their institutional review boards.^8^ This study followed the Consolidated Standards of Reporting Trials (CONSORT) reporting guideline for cluster trials.^28^

### Study Measures

The GAP70+ trial collected 27 PRO-CTCAE symptom items with multiple attributes.^2,8,18^ The investigators selected those items based on their relevance to older adults when designing the trial in 2013.^18^ For this analysis, we included 24 PRO-CTCAE items with severity attributes collected before treatment. Severity responses for each item range from 0 to 4, corresponding to none, mild, moderate, severe, and very severe.^14^ A total severity score was calculated as the sum of 24 items (range, 0-96).

The primary outcome of this study was whether a participant experienced unplanned hospitalization(s) within 3 months of starting a new treatment regimen. Any planned or scheduled admissions were excluded from the analysis. The secondary outcomes included all-cause mortality over 1 year of study enrollment and any grade 3 to 5 toxic effects within 3 months. Data on hospitalization and date of death were captured by practice staff. All clinician-reported grade 3 to 5 toxic effects were prospectively collected and reviewed using the NCI CTCAE version 4.^8^ Clinic notes and discharge summaries were reviewed by blinded clinicians at the URCC NCORP Research Base, and the treating physician was queried if there was any discrepancy. Detailed sociodemographic information, cancer diagnosis and stage, cancer treatments, Karnofsky performance status (KPS), and 8 GA domains (eg, polypharmacy, functional status, comorbidity) were collected as described previously.^8,18^ Race (American Indian or Alaskan Native, Asian, Black or African American, Native Hawaiian or other Pacific Islander, and White) and ethnicity (Hispanic or Latino, non-Hispanic, and unknown) was self-reported by patients. Race and ethnicity information was collected in the original trial to capture the diversity of trial enrollees. We collapsed racial and ethnic groups into non-Hispanic Black, non-Hispanic White, and other due to small sample sizes.

### Statistical Analysis

Our first step grouped patients into clusters with similar mixes of symptom severity using an unsupervised machine learning algorithm (k-means with Euclidean distance). We selected k-means because of its computational efficiency and intuitive visualization of the data points related to their respective clusters.^23^ The k-means algorithm clustered severity attributes of 24 PRO-CTCAE items that patients provided at regimen initiation; k-means is a centroid-based clustering algorithm that performs this grouping by partitioning a data set into *k* clusters by minimizing the sum of squared distance in each cluster.^23,29,30^ At each step, there are 3 main steps in the process. First, a number of clusters (*k*) was specified, and then the k-means algorithm randomly selected k patients as the initial cluster centers.^29,30^ Second, the algorithm assigned each patient to the closest centroid, and the cluster centroid was updated sequentially. Finally, this process was repeated until the total within the sum of square was minimized and each patient was assigned to 1 cluster based on the distance to the centers, measured by the Euclidean distance.^23,30^ The number of clusters was determined by the visual examination of the reduction in the sum of squared distances by the changes in clusters. A visual presentation of the clustering was presented. During the entire clustering process, the algorithm was blinded to outcome variables.

After establishing symptom severity clusters, we compared sociodemographic and clinical variables as well as outcome measures by cluster. To examine the validity of the clustering, we compared the severity attributes of individual items and total severity score by cluster. Differences across clusters were tested using analysis of variance for continuous variables and χ^2^ tests for binary variables. To determine whether symptom clusters were associated with adverse outcomes (hospitalization, mortality, and toxic effects), we conducted both unadjusted and adjusted analyses. First, for each outcome, we built a model with the outcome as the dependent variable, symptom clusters as the fixed effect, and practice sites as random effects (unadjusted analysis). In the second step, we further controlled for study group and additional predetermined sociodemographic and clinical factors that might be associated with outcomes.^2,8^ These factors included age, sex, cancer type, cancer treatment, number of GA domain impairments, and KPS.^2^ For hospitalization, we performed generalized linear mixed models (GLMM) with binary distribution and log link and reported risk ratios and adjusted risk ratios as measures of the association of symptom clusters with the outcomes. We examined the association of symptom clusters with 1-year all-cause mortality using the Cox shared frailty model, adjusting for practice sites as random effects^8,31^ and reported hazard ratios and adjusted hazards ratios. A Kaplan-Meier plot was created to contrast the 1-year survival by cluster. We conducted multivariable logistic regression on toxic effects because the adjusted GLMM model with practice sites did not converge.

Clustering was implemented using the Cluster package in R version 4.0 (R Project for Statistical Computing). The remaining statistical analyses were performed in SAS version 9.4 (SAS Institute) and Stata version 16.0 (StataCorp), with 2-tailed *P* < .05 to establish the statistical significance.

## Results

Of the 706 older adults included in the analysis (eFigure in Supplement 2), the mean (SD) age was 77.2 (5.5) years, and most patients were non-Hispanic white (619 [87.8%]; 51 [7.2%] Black patients), received at least a high school education (597 [84.6%]), and were married or in a domestic partnership (443 [62.7%]) (Table 1). Our sample included 401 male patients (56.8%) and 305 female patients (43.2%). Gastrointestinal, lung, and genitourinary cancers accounted for 34.7% (245 patients), 24.8% (175 patients), and 15.4% (109 patients) of the sample; most patients were diagnosed as stage IV (617 [87.4%]). Most patients (621 [88.0%]) planned to receive chemotherapy, while the remaining patients were on targeted therapy or hormonal therapy. The mean (SD) number of impaired GA domains was 4.5 (1.6), with the top 3 impaired domains being physical performance (657 [93.1%]), polypharmacy (572 [81.0%]), and comorbidity (475 [67.3%]). Over 3 months after baseline, 178 patients (25.2%) experienced unplanned hospitalization. More than 40% of the patients died within 1 year of enrollment (337 [47.7%]) and more than half reported any toxic effect in 3 months (436 [61.8%]). Table 2 describes the symptom burden among the patients enrolled. The mean (SD) total severity score was 13.97 (9.27).

**Table 1.** Patient Characteristics by Symptom Severity Clusters

Factors	Patients, No. (%)				*P* value^a^
	All (N = 706)	Severity cluster			
		Low (n = 310)	Moderate (n = 295)	High (n = 101)	
Age, mean (SD), y	77.20 (5.45)	77.68 (5.42)	76.91 (5.39)	76.56 (5.63)	.01
Sex					
Male	401 (56.8)	190 (61.3)	159 (53.9)	52 (51.5)	.09
Female	305 (43.2)	120 (38.7)	136 (46.1)	49 (48.5)	
Race and ethnicity^b^					
African American or Black	51 (7.2)	18 (5.8)	21 (7.1)	12 (11.9)	.25
Non-Hispanic White	619 (87.8)	277 (89.6)	260 (88.1)	82 (81.2)	
Other^c^	35 (5.0)	14 (4.5)	14 (4.7)	7 (6.9)	
Education					
<High school	109 (15.4)	40 (12.9)	46 (15.6)	23 (22.8)	.12
High school graduate	239 (33.9)	101 (32.6)	107 (36.3)	31 (30.7)	
≥Some college	358 (50.7)	169 (54.5)	142 (48.1)	47 (46.5)	
Marital status					
Single, never married	16 (2.3)	8 (2.6)	7 (2.4)	1 (1.0)	.13
Married or domestic partnership	443 (62.7)	200 (64.5)	171 (58.0)	72 (71.3)	
Separated, widowed, or divorced	247 (35.0)	102 (32.9)	117 (39.7)	28 (27.7)	
Cancer type					
Breast	55 (7.8)	25 (8.1)	23 (7.8)	7 (6.9)	.05
Gastrointestinal	245 (34.7)	99 (31.9)	102 (34.6)	44 (43.6)	
Genitourinary	109 (15.4)	60 (19.4)	38 (12.9)	11 (10.9)	
Gynecological	41 (5.8)	15 (4.8)	21 (7.1)	5 (5.0)	
Lung	175 (24.8)	68 (21.9)	81 (27.5)	26 (25.7)	
Lymphoma	46 (6.5)	29 (9.4)	12 (4.1)	5 (5.0)	
Others	35 (5.0)	14 (4.5)	18 (6.1)	3 (3.0)	
Cancer stage					
Stage III (palliative intent)	76 (10.8)	33 (10.6)	33 (11.2)	10 (9.9)	.14
Stage IV	617 (87.4)	274 (88.4)	257 (87.1)	86 (85.1)	
Other	13 (1.8)	3 (1.0)	5 (1.7)	5 (5.0)	
Cancer treatment					
Single chemotherapy agent	145 (20.5)	68 (21.9)	64 (21.7)	13 (12.9)	.045
Multiple chemotherapy agents	327 (46.3)	126 (40.6)	141 (47.8)	60 (59.4)	
Chemotherapy and other agents	149 (21.1)	72 (23.2)	59 (20.0)	18 (17.8)	
Other treatment (hormonal, and targeted)	85 (12.0)	44 (14.2)	31 (10.5)	10 (9.9)	
Karnofsky performance status^b^					
20-60	89 (12.6)	21 (6.8)	46 (15.6)	22 (21.8)	<.001
70-80	373 (52.9)	144 (46.5)	168 (57.1)	61 (60.4)	
90-100	243 (34.5)	145 (46.8)	80 (27.2)	18 (17.8)	
Geriatric Assessment domains					
Impaired domains, mean (SD), No.	4.50 (1.57)	3.80 (1.40)	4.89 (1.46)	5.49 (1.44)	<.001
Polypharmacy	572 (81.0)	240 (77.4)	246 (83.4)	86 (85.1)	.09
Functional status	404 (57.2)	137 (44.2)	193 (65.4)	74 (73.3)	<.001
Physical performance	657 (93.1)	280 (90.3)	279 (94.6)	98 (97.0)	.03
Comorbidity	475 (67.3)	180 (58.1)	209 (70.8)	86 (85.1)	<.001
Nutrition	427 (60.5)	139 (44.8)	207 (70.2)	81 (80.2)	<.001
Medical social support	190 (26.9)	67 (21.6)	94 (31.9)	29 (28.7)	.02
Psychological status	196 (27.8)	38 (12.3)	101 (34.2)	57 (56.4)	<.001
Cognition	254 (36.0)	98 (31.6)	113 (38.3)	43 (42.6)	.08
Outcomes					
Unplanned hospitalization in 3 mo	178 (25.2)	56 (18.1)	87 (29.5)	35 (34.7)	<.001
Deceased in 1 y	337 (47.7)	117 (37.7)	153 (51.9)	67 (66.3)	<.001
Any grade 3-5 toxic effect	436 (61.8)	185 (59.7)	184 (62.4)	67 (66.3)	.47

^a^ *P* values measure whether patient characteristics differed by severity clusters, using analysis of variance for continuous variables and χ^2^ tests for binary variables.

^b^ Missing data were only found for race and ethnicity (1 participant) and Karnofsky performance status (1 participant).

^c^ Other included Asian, American Indian or Alaskan Native, Native Hawaiian or other Pacific Islander, and Hispanic.

**Table 2.** PRO-CTCAE Items by Symptom Severity Clusters

Pro-CTCAE items	Mean (SD)				*P* value^a^
	All patients (N = 706)	Severity cluster			
		Low (n = 310)	Moderate (n = 295)	High (n = 101)	
Total severity score	13.97 (9.27)	6.33 (3.44)	16.57 (4.32)	29.80 (7.80)	<.001
Fatigue	1.60 (1.07)	0.79 (0.75)	2.03 (0.73)	2.82 (0.79)	<.001
Pain	1.18 (1.10)	0.61 (0.82)	1.49 (1.03)	2.05 (1.14)	<.001
Decreased appetite	1.16 (1.19)	0.37 (0.66)	1.48 (1.03)	2.68 (1.01)	<.001
Insomnia	0.97 (1.07)	0.45 (0.68)	1.17 (1.03)	2.01 (1.20)	<.001
Shortness of breath	0.95 (1.09)	0.51 (0.74)	1.21 (1.14)	1.52 (1.31)	<.001
Constipation	0.81 (1.09)	0.40 (0.76)	0.87 (1.00)	1.91 (1.36)	<.001
Dry mouth	0.74 (0.97)	0.32 (0.62)	0.78 (0.85)	1.90 (1.17)	<.001
Diarrhea	0.71 (0.92)	0.39 (0.62)	0.89 (0.99)	1.15 (1.12)	<.001
Problems with memory	0.63 (0.81)	0.33 (0.55)	0.78 (0.81)	1.14 (1.05)	<.001
Numbness or tingling	0.58 (0.92)	0.35 (0.72)	0.68 (0.94)	0.94 (1.19)	<.001
Problem tasting	0.55 (0.99)	0.07 (0.28)	0.57 (0.92)	1.94 (1.25)	<.001
Nausea	0.48 (0.79)	0.14 (0.41)	0.56 (0.74)	1.33 (1.06)	<.001
Arm or leg swelling	0.47 (0.86)	0.23 (0.59)	0.61 (0.94)	0.83 (1.07)	<.001
Headaches	0.46 (0.73)	0.24 (0.55)	0.58 (0.78)	0.75 (0.90)	<.001
Problems with concentration	0.45 (0.76)	0.16 (0.44)	0.49 (0.70)	1.24 (1.05)	<.001
Dizziness	0.42 (0.74)	0.15 (0.43)	0.52 (0.78)	0.98 (0.97)	<.001
Blurry vision	0.36 (0.74)	0.18 (0.53)	0.38 (0.76)	0.84 (1.01)	<.001
Ringing in ears	0.35 (0.73)	0.28 (0.65)	0.34 (0.69)	0.59 (0.99)	<.001
Difficulty swallowing	0.25 (0.65)	0.06 (0.35)	0.23 (0.59)	0.89 (1.05)	<.001
Hair loss	0.23 (0.68)	0.12 (0.43)	0.27 (0.76)	0.45 (0.92)	<.001
Vomiting	0.20 (0.55)	0.04 (0.22)	0.21 (0.50)	0.72 (0.94)	<.001
Mouth and throat sores	0.16 (0.54)	0.05 (0.30)	0.16 (0.46)	0.53 (0.98)	<.001
Hand foot syndrome	0.14 (0.48)	0.06 (0.28)	0.16 (0.51)	0.38 (0.75)	<.001
Skin cracking at corners of mouth	0.12 (0.45)	0.03 (0.16)	0.13 (0.41)	0.40 (0.85)	<.001

Abbreviation: PRO-CTCAE, Patient-Reported Outcomes version of the Common Terminology Criteria for Adverse Events.

^a^ *P* values measure whether PRO-CTCAE severity items differed by severity clusters using analysis of variance.

**Figure 1.**
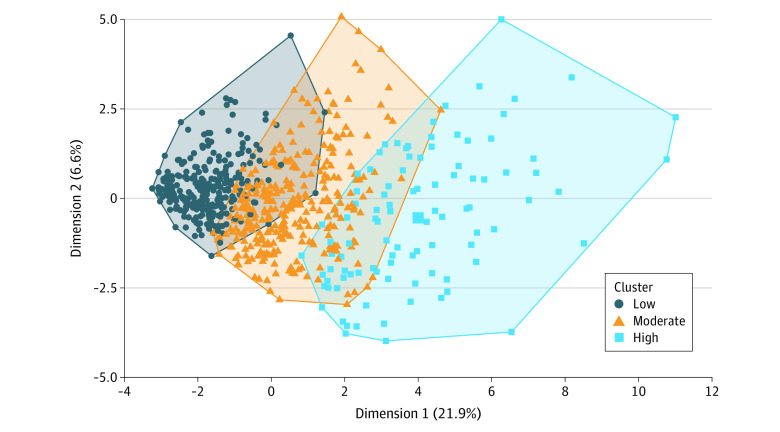
Clustering of Patient-Reported Outcome Version of the Common Terminology Criteria for Adverse Events Severity Items Three clusters were found using the k-means method with Euclidean distance. For the purpose of data visualization, the x- and y-axes are principal components of the 24 PRO-CTCAE severity items.

**Figure 2.**
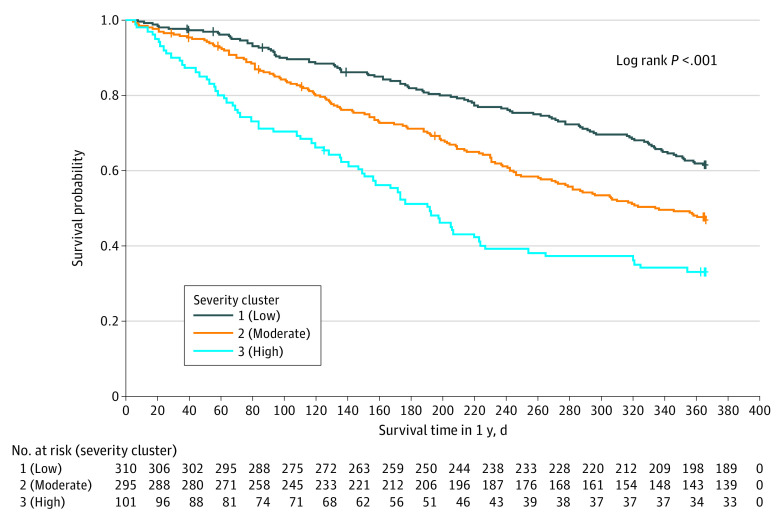
One-Year Overall Survival by Symptom Severity Cluster Three severity clusters were identified from the 24 severity items in the Patient-Reported Outcomes version of the Common Terminology Criteria for Adverse Events, using k-means method with Euclidean distance.

Figure 1 visualizes the symptom severity clusters identified by the k-means algorithm. The algorithm classified 310 patients (43.9%; dark blue green dots), 295 (41.8%; orange dots), and 101 (14.3%; light blue dots) into low-, medium-, and high-severity clusters (within-cluster mean [SD] severity score: low-severity, 6.33 [3.44]; moderate-severity 16.57 [4.32]; high-severity, 29.80 [7.80]; *P* < .001) (Table 2). Compared with patients in the low-severity cluster, patients in the moderate- and high-severity clusters also reported higher severity for all individual symptom items. No major difference in sociodemographic factors or cancer diagnosis was found among clusters. Patients in the low-severity cluster were slightly older than those in the moderate and high clusters (mean [SD] age: low, 77.7 [5.4] years; moderate, 76.9 [5.4] years; high, 76.6 [5.6] years). Patients in the high-severity cluster were more likely to receive multiple chemotherapy agents and have poorer KPS scores and more GA impairments (Table 1). The percentage of patients who were hospitalized was 18.1% (59 patients), 29.5% (87 patients), and 34.7% (35 patients) in the low-, moderate-, and high-severity clusters (*P* < .001). Similarly, patients in the moderate- and high-severity clusters were at a higher risk of death and toxic effects. Figure 2 shows that patients in the moderate- and high-severity clusters had an elevated risk of death from the beginning of follow-up time.

Table 3 presents both unadjusted and adjusted regression results for outcome measures. In the unadjusted model, compared with patients in the low-severity cluster, those in the moderate– and high–symptom severity clusters were significantly more likely to experience hospitalization (moderate: risk ratio, 1.61 [95% CI, 1.20-2.17]; *P* = .02; high: risk ratio, 1.88 [95% CI, 1.32-2.69]; *P* < .001) and at a higher risk of death (moderate: hazard ratio, 1.59 [95% CI, 1.23-22.03]; *P* < .001; high: hazard ratio, 2.62 [95% CI, 1.93-.3.53]; *P* < .001). Patients in the moderate– and high–symptom severity clusters were not significantly associated with a high risk of toxic effects. After controlling for predetermined covariates, the association of symptom clusters and outcome measures became weaker but remained significant for hospitalization and death. Specifically, compared with patients in the low-severity cluster, patients in the moderate-severity cluster were more likely to experience hospitalization (adjusted risk ratio, 1.36 [95% CI, 1.01-1.84]; *P* = .046) but not patients in the high-severity cluster (adjusted risk ratio, 1.44 [95% CI, 0.99-2.10]; *P* = .05). Similarly, compared with the low-severity cluster, the moderate- and high-severity clusters were associated with a higher risk of death (moderate: adjusted hazard ratio, 1.31 [95% CI, 1.01-1.69]; *P* = .04; high: adjusted hazard ratio, 2.00 [95% CI, 1.43-2.78]; *P* < .001). The association of moderate- and high-severity clusters with toxic effects was not statistically significant (moderate: adjusted hazard ratio, 1.04 [95% CI, 0.92-1.18]; *P* = .49; high: adjusted hazard ratio, 1.17 [95% CI, 0.97-1.39]; *P* = .10).

**Table 3.** Associations of Symptom Severity Clusters With Longitudinal Adverse Outcomes

Variable	Hospitalization over 3 mo, RR (95% CI)		Overall survival over 1 y, HR (95% CI)		Any grade 3-5 toxic effect, RR (95% CI)	
	Unadjusted model^a^	Adjusted model^a^^,b^	Unadjusted model^a^	Adjusted model^a^^,^^b^	Unadjusted model^a^	Adjusted model^b^^,^^c^
Symptom cluster, severity						
Low	1 [Reference]	1 [Reference]	1 [Reference]	1 [Reference]	1 [Reference]	1 [Reference]
Moderate	1.61 (1.20-2.17)	1.36 (1.01-1.84)	1.59 (1.25-22.03)	1.31 (1.01-1.69)	1.05 (0.92-1.19)	1.04 (0.92-1.18)
*P* value	.02	.046	<.001	.04	.50	.49
High	1.88 (1.32-2.69)	1.44 (0.99-2.10)	2.62 (1.93-3.53)	2.00 (1.43-2.78)	1.11 (0.94-1.31)	1.17 (0.97-1.39)
*P* value	<.001	.05	<.001	<.001	.21	.10
Patients, No.	706	706	706	706	706	706
Sites, No.	39	39	39	39	39	NA

Abbreviations: HR, hazard ratio; NA, not applicable; RR, risk ratio.

^a^ Models included practice site as random effect.

^b^ Multivariable models adjusted for age, sex, cancer type, cancer treatment, number of Geriatric Assessment domain impairments, study group, Karnofsky performance status.

^c^ Practice site random effect was excluded because the model did not converge.

## Discussion

In this secondary analysis of a large national randomized trial of more than 700 older adults with advanced cancer receiving active treatment, we identified 3 main symptom clusters (low, moderate, and high severity) using a clustering algorithm. The symptom severity score of patients in each cluster differed. Patients in moderate- and high-severity clusters at baseline were more likely to experience unplanned hospitalization and have worse overall survival but had similar risks of toxic effects. Our results demonstrate that unsupervised machine learning methods can be used to identify patients who are at higher risk for adverse outcomes based on their baseline patient-reported symptoms.

Unsupervised machine learning proves to be an effective bottom-up method of stratifying patients into distinct symptom severity clusters. Both the total severity score and all individual items were higher in the moderate- and high-severity clusters compared with those in the low-severity cluster, confirming the validity of the k-means algorithm. Compared with traditional methods of determining patient subgroups, k-means is more flexible and does not need human input (eg, determining the cutoff values). Another strength of the unsupervised algorithm is that no outcome measure is needed to identify the clusters.

A simple algorithm that does not require large computational resources, k-means has promising potential in clinical research and practice.^20^ Researchers can use k-means as a dimension reduction tool in research, in which the symptom clusters identified can be used as factors in traditional regression methods. Health systems can build k-means algorithm into existing electronic health record systems for clinical decision aid.^32^ When properly implemented and validated, clinicians can effectively identify symptom patterns for older adults before their cancer treatments. This summarized information can be used with GA to assess the vulnerability of older adults.^2^ For patients in the high-severity cluster, clinicians may consider modifying treatment options and monitor adverse events during and after treatments.^20^ Clinical studies are warranted to examine whether clinicians use the clustering information to inform treatment decisions and improve patient outcomes, similar to recent GA trials.^8,33^

A major finding of this study is that older adults with advanced cancer in the moderate- and high-severity clusters were more likely to experience unplanned hospitalization. This finding is consistent with prior literature that observed an association between higher baseline symptom burden and adverse outcomes in patients with cancer.^34,35^ A population-based study of patients with head and neck cancer found an association between patient-reported symptom burden and subsequent emergency department use and unplanned hospitalization.^35^ Another recent study demonstrated an association between symptom burden and impairment in physical function among older adults with cancer.^36^ In contrast to our study, neither study conducted a cluster analysis assessing the association between symptom burden and hospitalization. Studies using clustering methods often have not focused on older adults or examined longitudinal outcomes.^37,38^

Our results also suggest that moderate to severe baseline symptom severity are independently associated with all-cause mortality in older adults with advanced cancer receiving palliative systematic treatment. This suggests that assessment of symptom burden prior to treatment initiation contributes clinically useful prognostic information for treatment decision-making among older adults. Because of the expected limited survival in this population, baseline symptom burden could be more clinically meaningful for estimating outcomes than the response to treatment, the traditional oncology clinical trial end point.^39^

We hypothesize 3 possible mechanisms to explain the higher risks of hospitalization and death among patients in the moderate- and high-severity clusters. First, patients in the moderate- and high-severity clusters had more symptomatic burden that was not fully captured by GA and KPS, contributing to their vulnerabilities. These patients could potentially benefit from palliative care during their cancer treatments to reduce their symptom burden and improve their quality of life.^11,40^ Second, higher baseline symptoms were associated with more aggressive cancers (eg, pancreatic cancer) and the receipt of multiple chemotherapy regimens, which may reflect the fact that patients with severe symptoms had more aggressive disease progression and therefore needed multiple agents. The third possible mechanism is the elevated risk of toxic effects from the cancer treatment. Patients in the moderate- and high-severity clusters had slightly higher risk ratios for the probability of clinician-rated grade 3 to 5 toxic effects than those in the low-severity cluster, but the associations were not statistically significant. Those findings suggest treatment-related toxic effects are a potential but weak pathway toward hospitalization and death.

Our study has clinical and public health implications. First, unsupervised machine learning may offer an option to obtain summary information from PRO-CATE, for which no total score was available. Second, the association between symptom severity at the initiation of a new palliative regimen and adverse outcomes among older adults with advanced cancer suggests that PRO-CTCAEs can provide information in addition to GA and KPS. The inclusion of patient-centered assessment tools, such as PRO-CTCAEs, in clinical practice can assist clinicians in treatment decision-making and supportive care recommendations.^2^ Moreover, machine learning algorithms could identify patients with advanced cancer who are at a higher risk of poor treatment tolerability and short-term mortality.

### Limitations

Our study has limitations. Patients included were mostly non-Hispanic White with high levels of education; they do not represent the entire US population. The k-means algorithm is sensitive to outliers in the clustering variables, although all clustering variables ranged from 0 to 4. Euclidean distances might be distorted by the potential collinearity among the clustering variables. We only included PRO-CTCAE severity attributes; future analyses should expand to other attributes. Additionally, our k-means algorithm needs external validation.

## Conclusions

In this secondary analysis of a cluster trial of more than 700 older adults, unsupervised machine learning effectively identified patients with similar characteristics and stratified patients into clusters based on symptom severity. Our findings reinforce the importance of routine symptom assessment prior to treatment initiation as a best practice standard. PRO-CTCAEs assessed at treatment initiation can add information to the GA and performance status on patient vulnerability and inform potential treatment tolerability. Machine learning may be used to guide the development of risk stratification tools with the potential to assist clinicians in identifying older adults with a high risk of adverse outcomes.

## Notes

Supplement 1.
Trial Protocol

Supplement 2. **eFigure.** Study Flow Diagram

Supplement 3.
Data Sharing Statement
