# Supplementary material for: An Unsupervised Machine Learning Approach to Evaluating the Association of Symptom Clusters With Adverse Outcomes Among Older Adults With Advanced Cancer: A Secondary Analysis of a Randomized Clinical Trial
Source: JAMA Netw Open. 2023 Mar 22;6(3):e234198. doi: 10.1001/jamanetworkopen.2023.4198 (PMC10034574; doi:10.1001/jamanetworkopen.2023.4198)
Supplement: Supplement 1. — Trial Protocol [file jamanetwopen-e234198-s001.pdf]

A Geriatric Assessment Intervention for Patients Aged 70 and Over Receiving Chemotherapy or Similar Agents for Advanced Cancer: Reducing Toxicity in Older Adults

Protocol #: URCC13059

URCC / University of Rochester NCORP Research Base
NCT02054741

**Study Chairs:**	Supriya Mohile, MD, MS<br>James P. Wilmot Cancer Institute<br>URCC NCORP Research Base<br>601 Elmwood Avenue, Box 704<br>Rochester, NY 14642<br>(585) 275-5513
**Co-Principal Investigators**	William Dale, MD, PhD<br>Arti Hurria, MD
**Research Base Co-Chairs**	Gary Morrow, PhD, MS<br>Karen Mustian, PhD, MPH
**Co-investigators**	Gary Morrow, PhD, MS<br>Karen Mustian, PhD, MPH<br>Marie Flannery PhD, RN AOCN<br>Ronald Epstein, MD<br>David Dougherty, MD<br>Ekaterina (Katia) Noyes, PhD<br>Mohamed Tejani, MD<br>Arti Hurria, MD<br>William Dale, MD, PhD<br>Judith Hopkins, MD<br>Rakesh Gaur, MD
**Statistics Co-Chair**	Charles Heckler, PhD

Protocol submitted to NCI: 20-Jun-13
Protocol submitted to NCI: 13-Sept-13
NCI approved: 1-Oct-13
Amendment submitted to NCI: 13-Dec-13
Revised Amendment submitted to NCI: 23 Dec-13
NCI approved: 15-Jan-14
Amendment submitted to NCI: 3-Feb-14
NCI approved: 10-Feb-14
Amendment submitted to NCI: 17-March-14
NCI approved: 19-March-14
Amendment submitted to NCI: 13-Oct-14
NCI approved 27-Oct-14

PROTOCOL CONTACT INFORMATION

URCC NCORP Research Base:

URCC NCORP Research Base
Saunders Research Building
265 Crittenden Blvd
Box CU 420658Rochester, NY 14642

phone: 585-275-5513
fax: 585-461-5601
website: http://extranet.urmc.rochester.edu/NCORP/

Principal Investigators:

Supriya Mohile, MD, MS
URCC NCORP Research Base
William Dale, MD, PhD
University of Chicago
Arti Hurria, MD
City of Hope Cancer Center

Co-Investigators:

Gary Morrow, PhD, MS
Karen Mustian, PhD, MPH
Marie Flannery, PhD, RN, AOCN
URCC NCORP Research Base
Ronald Epstein, MD
David Dougherty, MD
Ekaterina (Katia) Noyes, PhD
Mohamed Tejani, MD
University of Rochester

Judith Hopkins, MD
SCCC NCORP Community Site
Rakesh Gaur, MD
Kansas City NCORP Community Site

Biostatistician

Charles Heckler, PhD
URCC NCORP Research Base

Regulatory Contact:

Cathy Lesniewski
585-275-2282
cathleen_lesniewski@urmc.rochester.edu

Data Contact:

Libby Nagalski
585-275-1364
elizabeth_nagalski@urmc.rochester.edu

Program Managers:

Nikesha J. Gilmore, PhD
585-275-1275
nikesha_gilmore@urmc.rochester.edu
Megan McKillip, MPH
585-275-1277
megan_mckillip@urmc.rochester.edu

Senior Program Manager:

Sandy Plumb
585-324-4553
sandy_plumb@urmc.rochester.edu

TABLE OF CONTENTS

STUDY SCHEMA AND SUMMARY 3
1. BACKGROUND INFORMATION 4
2. STUDY OBJECTIVES 9
3. STUDY DESIGN 11
4. PARTICIPANT ELIGIBILITY 11
5. IDENTIFICATION, RECRUITMENT, AND CONSENT PROCEDURES 12
6. REGISTRATION 14
7. OUTCOMES 16
8. MEASURES TO BE COLLECTED 18
9. NCORP PRACTICE SITE RANDOMIZATION 21
10. INTERVENTION OVERVIEW FOR PHYSICIANS 22
11. OUTLINE OF STUDY-SPECIFIC PROCEDURES 23
12. REIMBURSEMENT 29
13. ADVERSE EVENT REPORTING 29
14. CRITERIA FOR WITHDRAWAL 31
15. STATISTICAL PLAN 32
16. DATA MANAGEMENT AND QUALITY ASSURANCE 34
17. DATA COLLECTION AND MEASURES 36
18. PARTICIPANT CONSENT AND PEER JUDGMENT 36
19. RECORD AND DATA RETENTION 36
20. REFERENCES 37

APPENDIX A: SUMMARY OF MEASURES
APPENDIX B: PARTICIPANT MEASURES
APPENDIX C: CLINICAL RESEARCH ASSOCIATE MATERIALS
APPENDIX D: PHYSICIAN MEASURES
APPENDIX E: STUDY RELATED FORMS
APPENDIX F: APPROVED OTHER AGENTS WITH SIMILAR PREVALENCE OF TOXICITY TO CHEMOTHERAPY

STUDY SCHEMA AND SUMMARY

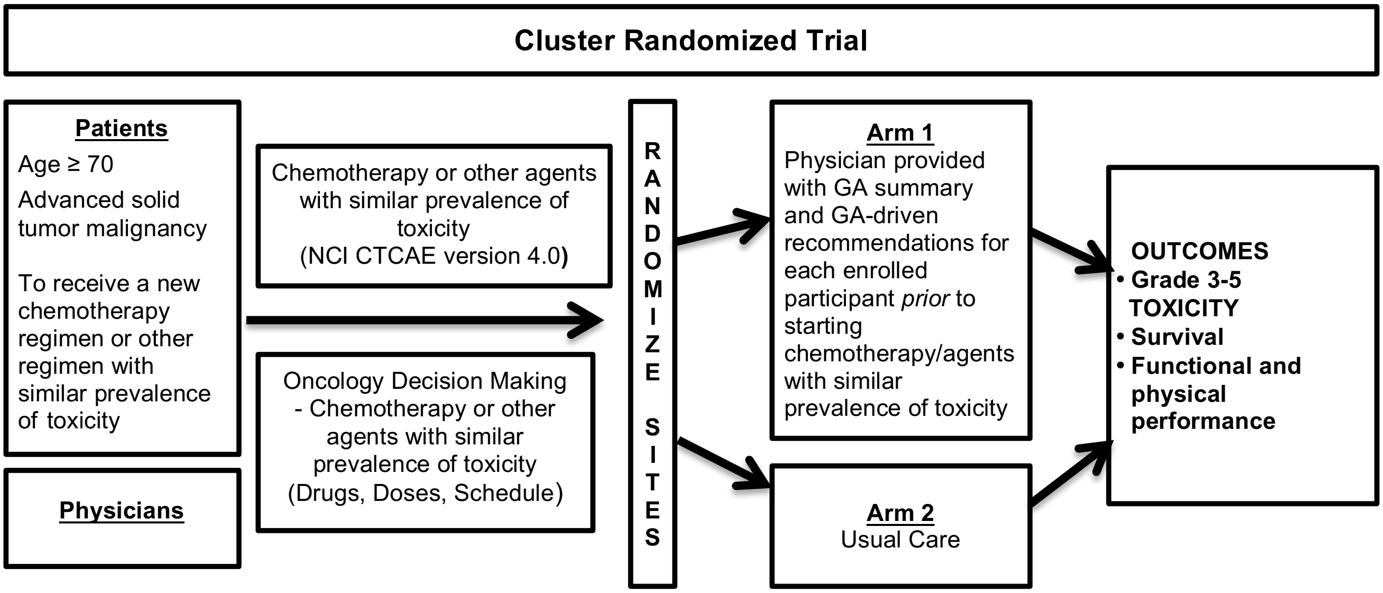

Adults age ≥70 who will start a new chemotherapy regimen or other regimen with similar toxicity prevalence (see section 4.2.1c) for an advanced solid tumor malignancy in the University of Rochester Cancer Center NCI Community Oncology Research Program (URCC NCORP) Research Base network will be eligible. Chemotherapy will be defined as cytotoxic drugs; in addition, agents (e.g., monoclonal antibodies and targeted agents) that have a prevalence of grade 3-5 toxicity in older patients similar to chemotherapy (>50%) will be allowed. Oncology physicians who practice at sites within the URCC NCORP Research Base network are participants in the study and will be enrolled. Their eligible patients will then undergo the informed consent process; those patients who agree to participate in this study will undergo a clinical assessment consisting of demographic characteristics and geriatric assessment (GA). All baseline assessments will be performed prior to initiation of the new treatment regimen.

NCORP practice sites with IRB approval of the protocol will be randomized to receipt of GA plus GA-driven recommendations (Arm 1) or usual care (Arm 2). A NCORP practice site will be defined as any practice location within an overarching NCORP designation where oncology physicians and study staff work independently (e.g., do not cross over into another site). In Arm 1, oncology physicians or their designees will be provided with GA summary plus targeted recommendations (i.e., GA-driven recommendations). GA-driven recommendations and the uptake of these recommendations along with the influence of the GA on decisions will be collected. In Arm 2, participants will complete the GA; but no GA summary or GA-driven recommendations will be provided to the oncology teams except for information regarding clinically significant cognitive impairment and/or depression. In both arms, participants will subsequently receive a treatment plan as prescribed by the treating oncology physician. Drugs and doses (throughout the entire course) will be recorded, as well as supportive care medications. NCI clinician-rated and patient-reported CTCAE grade 2-5 toxicities will be captured. In addition, dose delays, dose reductions, discontinuation of treatment, hospitalizations, and survival status will be captured, as well as the relationship of these events to toxicity. A brief follow-up GA will be collected at 4-6 weeks, 3 months, and 6 months after baseline registration. Survival will be captured for 1 year after study entry.

A total of 700 participants will be enrolled in this study. The acronym for this study is GAP70+, which stands for Geriatric Assessment for Patients 70+: A Bridge to Reduce Toxicities.

1. BACKGROUND INFORMATION

1.1. Intervention to be Studied

The URCC NCORP Research Base will conduct a cluster randomized study evaluating whether providing a GA summary with targeted recommendations (i.e., GA-driven recommendations) can lower toxicity in older adults receiving chemotherapy or other agents (e.g., monoclonal antibodies, targeted agents) ***with similar toxicity prevalence for advanced solid tumor malignancies***. Clinical outcomes will be captured including toxicity (both clinician and patient-reported), other adverse outcomes (e.g., hospitalizations, mortality), and changes in functional and physical performance.

1.2. Background and Significance

Although cancer is a disease of aging, older patients are underrepresented in clinical trials.[1,2] Balancing the benefits against the risks of chemotherapy in the older patient population is challenging because of the dearth of evidence-based data to guide these decisions.[3,4] Furthermore, older patients who are treated with chemotherapy are at high risk for adverse outcomes including serious chemotherapy toxicity and functional and physical consequences.[5-7] In addition, older patients have been found to be more susceptible to toxicity from other types of treatment (e.g., monoclonal antibodies and targeted agents) and some of these newer agents have similar prevalence of toxicity in older patients to chemotherapy.[117-120] For example, limited evidence exists to guide risks and benefits of the multi-kinase inhibitor, regorafenib, in the older adult. Participants in the Phase III CORRECT trial that led to approval of regorafenib in previously treated mCRC had a median age of 61. The small number of older participants (38 participants ≥ 75 years of a total n=503) in this study had a disproportionately higher risk of any ≥ grade 3 toxicities (66% for ≥ 75 years vs. 52% for < 65 years).[120]

The American Society of Clinical Oncology (ASCO) has stated that to improve quality of care, oncology physicians and patients should carefully weigh the risks and benefits of cancer-directed therapy for patients with a low performance status, who are not eligible for a clinical trial, and for whom there is no strong evidence supporting the clinical value of treatment.[8] These issues commonly affect older adults. The National Comprehensive Cancer Network (NCCN) guidelines advocate GA for older patients with cancer to identify health status issues that increase the risk of adverse outcomes.[9-13] A GA evaluates comorbidity, functional status, physical performance, cognitive ability, psychological status, medications and social support with standardized tools that predict morbidity and mortality in community-dwelling older adults.[14-16] Benefits of GA in community-dwelling older adults include prevention of geriatric syndromes, recognition of cognitive deficits, prevention of hospitalizations and nursing home admissions, and overall improvement of quality of life.[15,17-19] Evidence derived from research on GA-driven recommendations in community-dwelling older adults without cancer could benefit older patients with cancer, but this evidence has not been widely adopted by oncologists. The incorporation of GA as the standard of care in oncology has been slow due to lack of resources, difficulties with interpreting results, and difficulties with implementing targeted interventions.[12,20,21] Innovative and pragmatic interdisciplinary approaches to reduce risk of treatment in older cancer patients are imperative. This research supports the NIH commitment to trans-NIH strategic initiatives for the development of interdisciplinary research teams to address problems facing an aging nation.[22]

The overarching goal of this proposal is to evaluate whether providing the oncology team with information from the GA plus GA-driven recommendations can improve outcomes in older adults with advanced cancer. This proposal addresses the main objective of the U13 conference, "Geriatric Oncology Research to Improve Clinical Care," which is to develop innovative mechanisms to improve the clinical care of older cancer patients within the next 10 years.[3,4] The GA has great potential to identify areas of vulnerability and develop recommendations that could help improve outcomes (e.g., treatment toxicity) in older cancer patients.[14,15,23] The PI, in collaboration with Cancer and Aging Research Group (CARG) investigators, has found that older cancer patients have a high prevalence of characteristics that are

associated with a greater risk of chemotherapy toxicity.[24,25] This research is significant because: 1) a large and growing population of older cancer patients would benefit from the results; 2) GA information can identify risk factors for chemotherapy toxicity in older cancer patients; however, these questions are not routinely incorporated into the oncology clinical evaluation; and 3) a critical operational question exists: would provision of GA information along with GA-driven recommendations to the oncology treatment team improve outcomes in older patients with advanced cancer? Successful completion of the aims of this proposal will provide an innovative, pragmatic approach that could improve clinical care.

1.3. Condition to be Studied

A growing population of older patients is at high risk for adverse outcomes from cancer treatment. Cancer is a disease of aging; approximately 60% of all cancers and 70% of cancer mortality occur in persons aged 65 years and over.[26] The number of cancer patients over the age of 65 is projected to significantly increase over the next 20 years (see Figure 1).[27] Aging is a highly individualized process, characterized by an increased prevalence of health status conditions that can affect decision making for cancer treatment, treatment tolerance, and ultimately outcomes.[3,28,29] The PI has shown that older adults with cancer have a high prevalence of comorbidity, disability, and geriatric syndromes.[24,25] The majority of older patients with cancer are treated based on extrapolations of evidence derived from clinical trials providing data on the safety and efficacy of treatment in younger adults or in older patients who are fit without other health status conditions.[30] As a result, cancer treatment guidelines are often extrapolated from studies of younger, healthier patients. Patients with health status issues other than cancer are often excluded from oncology clinical trials, despite the fact that the majority of older adults have these issues at the time of cancer presentation.[25,31] Other health status issues can affect the ability to tolerate cancer therapy.[5,32-34]

Figure 1: Increase in older patients with cancer

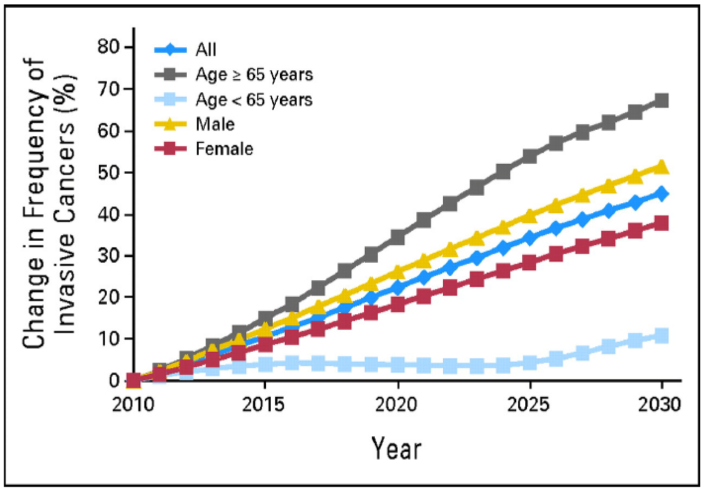

Pal S K , Hurria A JCO 2010;28:4086-4093

1.4. Geriatric Assessment (GA)

GA can identify risk factors for adverse outcomes in older cancer patients. Comprehensive GA includes a compilation of reliable, validated tools to assess geriatric domains such as comorbidity, functional status, physical performance, cognitive status, psychological status, nutritional status, medication review, and social support.[15,35-55] GA can detect unsuspected conditions that may affect cancer treatment in more than 50% of older patients.[56] Repetto et al. demonstrated that GA added information to standard oncology performance measures such as the Karnofsky score, a one-item measure of function, which was validated in younger patients. The GA has great potential to identify areas of vulnerability and interventions that could help improve outcomes (e.g., reducing treatment toxicity) in older cancer patients.[14,15,23] Notably, the primary investigator (PI) of this study, Dr. Supriya Mohile, in collaboration with CARG investigators, has demonstrated that older cancer patients have a high prevalence of characteristics that are associated with a greater risk of chemotherapy toxicity, and the GA can help identify these risk factors. Our research team has found the GA in this study was feasible in oncology clinics and trials.[5,12,23]

1.5 Geriatric Assessment Components and Relevance to the Older Patients with Cancer.

Currently, oncology physicians assess functional status by assigning a Karnofsky performance status score (KPS) or Eastern Cooperative Oncology Group (ECOG) performance status.[57,58] These generic scales are applied to all adult cancer patients, regardless of age, and are used to estimate functional status

in order to determine a treatment course, assess eligibility for clinical trials, and predict treatment toxicity and survival.[59,60] These tools may result in misleading decisions for the older patient, since clinical trials relying on these scales have largely excluded elderly patients. A prospective study of 500 older adults with cancer demonstrated that KPS could not identify older adults at risk for chemotherapy toxicity, while a predictive model including GA questions could identify such individuals.[5] The geriatrician's evaluation provides valuable information not provided by KPS or ECOG performance scores;[14,16,61,62] however, GA is not commonly taught in oncology training or utilized in oncology practice. A description of each GA domain and its relevance to the older patient with cancer is provided below.

1.5.1. Functional Status and Physical Performance. The need for functional assistance (measured by the ability to complete activities of daily living) is predictive of chemotherapy toxicity and survival.[63-66] Physical performance measures objectively evaluate mobility and fall risk.[43,53] Falls are common in older cancer patients and predictive of adverse outcomes.[5,25,67]

1.5.2. Comorbidity and Polypharmacy. Among patients with cancer, comorbidity is associated with poorer overall survival.[68-72] Comorbidity impacts cancer treatment tolerance.[73-76] Furthermore, these comorbid conditions may predispose patients to the risks of polypharmacy and drug interactions.[77]

1.5.3. Nutrition. Poor nutritional status is associated with an increased need for functional assistance and poorer overall survival in the geriatric population.[78] Unintentional weight loss during the six months prior to chemotherapy is associated with lower chemotherapy response rates and lower overall survival.[79]

1.5.4. Cognition. A cognitive assessment is needed to determine if the patient has the decisional capacity to consent and adhere to supportive care medication instructions and understand the indications to seek attention. In the presence of cognitive impairment, the involvement of the patient's family or caregiver is required to maintain safety.[80-83]

1.5.5. Psychological State and Social Support. In a study of older adults with cancer, significant distress was identified in 41% of older adults, and poorer physical function correlated with higher distress.[83] In both the geriatric and oncology literature, social isolation has been linked to an increased risk of mortality.[84-86]

1.6 Gap in Knowledge

A critical knowledge gap exists regarding which GA-driven recommendations have the greatest likelihood to improve outcomes of older cancer patients and how to implement these recommendations.[14,23,87] Despite the fact that the majority of cancer patients are in the older age groups, most oncologists have received little training in the care of older patients.[88] As a result, common problems facing an aging population of cancer patients may go unrecognized and have serious consequences.[87] For example, Hurria et al. revealed that although 5% of patients screened positively for severe cognitive impairment on GA, these patients were still consented onto a therapeutic protocol.[23] Thus, although GA predicts risk from chemotherapy toxicity, there is no evidence-based approach that demonstrates how to intervene to reduce risk from cancer treatment. An algorithm incorporating the best available evidence would help close the critical gap in knowledge regarding how to interpret GA results and implement interventions for older patients with cancer. The GA and GA-driven recommendations utilized in this proposal have been developed through preliminary work, extensive review of the evidence, and clinical expertise of the geriatric oncology physicians on the research team. Our research team has found that the GA in this study was feasible in oncology clinics and trials.[5,12,23]

1.7. Geriatric Assessment-Driven Recommendations and Relevance to Older Patients with Cancer

Interventions guided by GA have positive effects on health outcomes including the prevention of disability, and the reduction in the risk of falls, unplanned hospitalizations, and nursing home admissions, which provides evidence supporting the use of a multidimensional approach for older patients with cancer.[17-19] Several studies have shown that the implementation of GA and GA-driven recommendations into the clinical care of older patients with cancer is feasible.[12,21,56,89] The ELCAPA (elderly cancer patient) study illustrated that providing GA information and GA-driven recommendations to oncology teams can influence treatment decisions, although outcomes from these changes were not measured in this study.[89] This study showed that decisions for both chemotherapy and other regimens were affected by providing GA information to the oncology team. Another pilot study showed that GA affected the oncology treatment plan.[90] However, published randomized studies evaluating outcomes from GA and GA-driven recommendations in older cancer patients are scant. In a study by McCorkle et al.,[91] geriatric nurse practitioners conducted GA with cancer patients, and this led to a distinct survival advantage (67% in the intervention group compared with 40% in the control group). In a study by Goodwin et al., breast cancer patients in the GA-driven recommendations group were significantly more likely to return to normal functioning than the controls.[92] Different approaches for treatment selection and dosing for older and/or frail patients is supported by the literature and is incorporated into the framework as GA-driven recommendations (see Preliminary Data). The FOCUS-2 trial found that chemotherapy for advanced colorectal cancer was safe and efficacious in the older and/or frail patient if started at a 20% dose reduction with escalation as tolerated.[93]

1.8 Preliminary Data

The investigative team is poised to build upon a considerable body of prior work. The research team has conducted studies that have demonstrated the high prevalence of health status issues that could influence cancer outcomes in older patients.[24,25] They have developed a Geriatric Assessment tool for older persons with cancer.[12] The feasibility of this tool has been studied in hundreds of cancer patients in multicenter clinical trials.[5,12,23] They have collaborated on a prospective multicenter study to quantify the risks of chemotherapy among older adults with cancer.[5] Dr. Mohile has collected pilot data from over 200 patients from her referral-based geriatric oncology clinic which administers GA-driven recommendations.[94] Drs. Dale and Epstein have experience in the study of decision-making in oncology.

1.8.1. Prevalence of Health Status Issues in Older Patients with Cancer. Using a nationally representative population-based database, Mohile and collaborators (Dale and Morrow) published two investigations that demonstrated that disability, comorbidity, and geriatric syndromes (including falls) are more common in cancer patients and that cancer was independently associated with having these conditions (Figure 2).[24,25] In addition, Drs. Hurria and Mohile collected GA data from over 500 older cancer patients receiving chemotherapy at 7 institutions.[5] *The assessment revealed a number of findings that would not have been detected on routine history and physical exam:* 41% of patients needed assistance with instrumental activities of daily living despite a mean physician-reported KPS of 85, 16% had recent falls, and 6% had gross cognitive impairment on the cognitive screening test.

Figure 2: **Prevalence of Comorbidity, Disability, and Geriatric Syndromes**

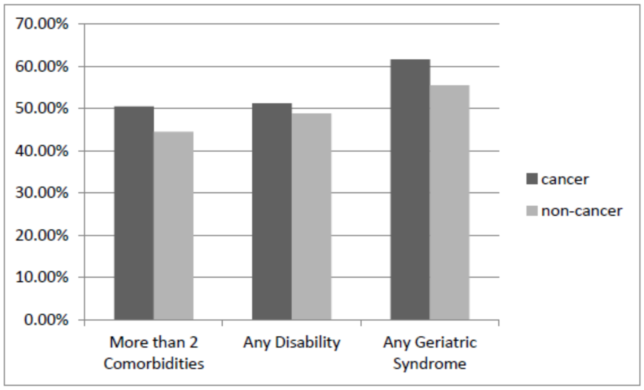

1.8.2. Developing a Geriatric Assessment for Older Adults with Cancer.[12] The geriatric and oncology literature was reviewed to choose validated GA measures. Selection criteria included reliability, validity, brevity, the ability to self-administer, and the ability to prognosticate risk for morbidity or mortality in an older patient.[12] The final selection of measures was approved by the Cancer and Leukemia Group B (CALGB) Cancer in the Elderly and Quality of Life Committees. The initial feasibility study of this tool was conducted in a multicenter study by Dr. Hurria and Dr. Mohile. Forty patients (mean age 74, range 65 to 87) with cancer participated in the study. The GA was feasible, as demonstrated by a mean time to completion of 27 minutes, 90% of participants were satisfied with the questionnaire length, and 78% were able to complete on their own.[12]

1.8.3. Feasibility of Geriatric Assessment Tool in Oncology Clinical Trials.[23] CALGB 360401 evaluated the feasibility of incorporating the GA into oncology cooperative group trials for older adults who had signed consent for a cooperative group treatment trial. Ninety-three patients enrolled in this study. The median time to complete the assessment was 22 minutes, 88% of participants completed the participant portion without assistance, 88% were satisfied with the assessment length, and 95% said the assessment was easy to comprehend. The GA for cancer patients met the protocol specified feasibility criteria for use in the cooperative group setting.

1.8.4. Can the Geriatric Assessment Predict Chemotherapy Toxicity?[5] The primary objective of this study was to determine if GA measures predicted grade 3-5 toxicity using the NCI Toxicity Index, Common Terminology Criteria for Adverse Events (CTCAE, V3.0). This study's eligibility was the same for the current proposed study: an older patient was enrolled prior to the start of a new chemotherapy regimen. Among the 500 enrollees, the mean age was 73 years (range 65-91). The most common tumor types were lung (29%), GI (29%) and breast/gynecologic (22%) cancers; 61% had metastatic disease and 71% received 1st line chemotherapy. Grade 3-5 toxicity occurred in 53% (50% grade 3, 12% grade 4, 2% grade 5). Risk factors for grade 3-5 toxicity included: 1) age ≥ 73, 2) cancer type (GI or GU), 3) standard dose, 4) poly-chemotherapy, 5) falls in last 6 months, 6) assistance with instrumental activities of daily living, and 7) decreased social activity. *In the published CARG study, 307 of 500 participants had advanced cancer and of these, 141 (46%) experienced grade 3, 4, or 5 toxicity within 3 months.*[5] *Seventy percent of the toxicities were non-hematologic (fatigue, nausea/vomiting, etc.).*

1.8.5. GA-driven interventions can influence oncology care and improve chemotherapy toxicity. Dr. Mohile directs a referral-based consultative geriatric oncology clinic which has collected pilot data on GA-driven interventions in over 200 participants.[94] Mean age was 82.1 (65-95) and 75% had advanced disease. GA revealed 68% with functional impairment, 70% had >3 significant comorbidities, 39% had poor nutrition,[95] 26% screened positive for depression,[96] 59% reported inadequate social support, 20% had an abnormal cognition screen,[97] 34% had recently fallen, and 60% had poor physical performance.[37,43] Dr. Mohile's research team prospectively evaluated the grade 3-5 toxicity rate of 100 consecutive participants that underwent GA and GA-driven recommendations. These participants were older (mean age 80, 70-91), but had similar cancer characteristics to the published observational cohort.[5] *Grade 3-4 toxicities occurred in 33 of 100 participants within 3 months of chemotherapy initiation. No participants developed grade 5 toxicities. This is lower than the rate reported in the CARG study of patients whose physicians did not receive GA results and did not implement GA-driven recommendations.*[5] *On average, 80% of the recommended GA-driven recommendations were implemented with an average of 6 recommendations per participant (range 3-15).* At the annual URCC Research Base meeting in September of 2012, site PIs expressed unanimous interest in the current proposal and >90% stated that they have the resources necessary to follow through with GA-driven recommendations (e.g., availability of PT/OT/Nutrition/Social work).

1.8.6. The research team has experience with interventions that affect decision-making for treatment of advanced cancer. Dr. Epstein, an expert in patient-centered communication, has used multi-method research to study patient-physician interactions involving analyses of patient and physician surveys and medical record audits. His research team has helped to establish that patient-centered communication is associated with improved information exchange, reduced symptom burden, lower heath care costs and greater patient involvement in decision-making.[98-100] The measures to assess decision-making in Dr. Epstein's NCI-funded RO1 (PI is a co-investigator) have been adapted for patients with advanced cancer.[101,102] Dr. Dale, Chief of Geriatrics and Palliative Care at the University of Chicago, has expertise in medical decision-making, quality of life, and frailty, and has studied the role of emotions in decisions about screening, diagnosis, and treatment of cancer in older persons. He and Dr. Mohile have collaborated on a study that evaluated patient-physician decisions with regard to treatment of advanced prostate cancer.[103] The patient and physician measures utilized in this proposal to capture decision-making for cancer treatment are adapted from this prior work.

1.9. Summary of Background, Innovation, and Public Health Significance.

Historically, oncology clinical trials have excluded older patients with other health conditions and have included only limited numbers of patients aged 70 and over.[14] Underrepresentation of older adults occurs in clinical trials examining the safety and efficacy of chemotherapy regimens as well as other treatments such as monoclonal antibodies and targeted agents.[117-120] Older cancer patients are at an increased risk of treatment complications, and there is no standard approach to implementing interventions to reduce toxicity. Common assessment instruments in oncology (e.g., Karnofsky performance status scores) do not address critical geriatric domains that predict morbidity and mortality in the older patients (e.g., functional status, comorbidity, social support). A geriatric assessment (GA) includes a compilation of reliable, validated tools to assess health status. Several studies, including a multicenter Cancer and Aging Research Group (CARG) trial, have demonstrated that items in a GA can predict severe chemotherapy toxicities in older cancer patients.[5] However, no consensus exists on how to translate information from the GA into targeted interventions that have the potential to prevent adverse outcomes (e.g., toxicity, functional decline, lower quality of life) for older cancer patients. The vast majority of oncology physicians have not adopted the use of GA and GA-driven recommendations for older adults with cancer, largely because of lack of knowledge on how to interpret GA results and on whether GA-driven recommendations can improve clinical outcomes.

1.10 . Study Participants

The study involves adult human subjects.

NCORP practice sites will be randomized within a 2-arm cluster randomized design utilizing NCORP practice sites as the unit of randomization. A NCORP practice site will be defined as any practice location within an overarching NCORP designation where oncology physicians and study staff work independently (i.e., do not cross over into another site).

Study participants will include:

• ***Oncology physicians*** at NCORP practice sites. Oncology physicians can work with designated staff in the clinic to carry through specific study procedures outlined in the protocol, but staff are not considered study participants and will not be enrolled.

• ***Participants*** will complete surveys. Participants will all have advanced cancer and various levels of functional status.

2. STUDY OBJECTIVES

This is a cluster randomized study within the URCC NCORP Research Base network evaluating whether

GA summary plus GA-driven recommendations can improve clinical outcomes in older adults starting a new treatment regimen (i.e., chemotherapy or other agents with similar prevalence of toxicity) for the current diagnosis of an advanced solid tumor malignancy (advanced cancer).

2.1. Primary Aim
Our primary aim is to determine if providing information regarding GA plus GA-driven recommendations to oncology physicians reduces clinician-rated **grade 3-5 toxicity** in participants aged 70 and over with advanced cancer starting a new treatment regimen. The regimen must include a chemotherapy drug or other agents that have similar prevalence of toxicity (see section 4.2.1c).

The primary efficacy endpoint for this study is the proportion of participants that experience grade 3-5 toxicity within 3 months of starting a new treatment regimen.

2.2. Secondary Aims

2.2.1. The principal secondary endpoint for this study is the proportion of participants that are **alive** at 6 months after study entry.

2.2.2. Additional efficacy measures will evaluate whether providing oncology physicians with information regarding GA summary and GA-driven recommendations influences clinical care of older patients receiving treatment for advanced cancer.

We will compare **treatment decisions** (as measured by relative dose intensity of the agents administered in the first cycle). We will also compare the number and type of **GA-driven recommendations** implemented for older participants starting a new treatment regimen for advanced cancer.

2.3. Exploratory Aims

2.3.1. An exploratory aim is to determine whether providing the oncology team with GA information and GA-driven recommendations can slow **functional and physical decline** in older patients with advanced cancer.

Functional status will be measured with self-reported Instrumental Activities of Daily Living score. Physical performance will be measured with the OARS Physical Health Subscale, and the Short Physical Performance Battery.

2.3.2. In collaboration with the National Cancer Institute, other exploratory aims will evaluate the role of patient-reported CTCAE in the clinical care of older adults receiving cancer treatment. These aims include the following:

2.3.2a. To examine the association between patient-reported symptoms (as measured by PRO-CTCAE) and geriatric domains (as measured by geriatric assessment).

2.3.2b. To compare PRO-CTCAE and physician-rated CTCAE in a sample of older patients receiving chemotherapy or other agents with similar prevalence of toxicity.

2.3.2c. To examine the association between PRO-CTCAE and treatment decisions.

2.3.2d. To examine the association between PRO-CTCAE and adverse outcomes (early discontinuation of chemotherapy or other agents with similar prevalence of toxicity, hospitalizations, and mortality).

3. STUDY DESIGN

See Study Schema and Design for summary.

3.1. Choice of Comparators

Because GA is not performed by community oncology physicians and this study ultimately will allow patients and oncology physicians to choose their cancer treatments, a usual care comparator arm is appropriate and will allow for the accurate and appropriate assessment of how the intervention can reduce toxicities. This study design is similar to previous studies that evaluated the impact of providing summarized Health Related Quality of Life (HRQoL) information to patients and oncology physicians on communication and outcomes. Usual care was the comparator arm in these cluster randomized trials.[104]

3.2. Choice of Study Design

The study is designed as a cluster randomized trial because a care of service model is applied to each participant by the oncology team. If a cluster randomized design were not undertaken, there would be contamination in that oncology physicians could choose the care of service model if they were exposed to participants randomized to both arms. Given rapid changes that can occur in oncology practice with new supportive care and treatment agents, it is important to compare outcomes in the same time frame as would be possible in a cluster randomized study design compared to a "pre" versus "post" intervention study design.

4. PARTICIPANT ELIGIBILITY

4.1. Entry Criteria for Oncology Physicians

Oncology physicians must work at a NCORP practice site with no plans to leave that NCORP practice site or retire at the time of enrollment into the study.

4.2. Entry Criteria for Patients

4.2.1. Inclusion Criteria for Patients

4.2.1a. Male or female 70 years of age or older.

4.2.1b. Diagnosis of an advanced solid tumor malignancy (advanced cancer) or lymphoma. In most situations, this would be a stage IV cancer. Patients with a diagnosis of stage III cancer or lymphoma are eligible if cure is not possible or anticipated. Clinical staging without pathological confirmation of advanced disease is allowed.

4.2.1c. Plan to start a new cancer treatment regimen within 4 weeks from time of baseline registration. The treatment regimen is up to the discretion of the treating oncology physician. The regimen must include a chemotherapy drug or other agents that have similar prevalence of toxicity.

Patients who will receive monoclonal antibody therapy or other cancer therapies (e.g., tyrosine kinase inhibitors) are eligible if the other agents present a prevalence of toxicity similar to chemotherapy. These therapies can be used in combination with chemotherapy, as a single agent, or in combination with each other.

* Chemotherapy will be defined as cytotoxic drugs; in addition, agents (e.g., monoclonal antibodies and targeted agents) that have a prevalence of grade 3-5 toxicity in older patients similar to chemotherapy (>50%) will be allowed. A list of allowable agents (single and in

combination) meeting this toxicity criteria will be available on the URCC NCORP Research Base website as part of the study materials. Given the rapidly changing landscape of new drugs for cancer, the study team led by the PI will update the list accordingly after reviewing the toxicity profile of new therapies. **If the potentially eligible participant is to receive an approved drug or regimen not on the list, contacting the URCC NCORP Research Base study team is required for approval prior to participant enrollment**.

Patients who are receiving approved cancer treatment in combination with radiation are eligible.

A patient may also be enrolled on a treatment trial and participate in this study, if all other inclusion and exclusion criteria are met.

4.2.1d. Plan to be on chemotherapy or other allowable treatment (as per 4.2.1c) for at least 3 months (minimum 70 days) and be willing to come in for study visits.

The plan for treatment should be for at least 3 months at time of study enrollment. The treatment can stop earlier during the study at the discretion of the physician and patient (e.g., due to progression as noted through imaging, toxicity, or patient preference).

4.2.1e. Have at least one geriatric assessment domain meet the cut-off score for impairment other than polypharmacy per Table 2.

4.2.1f. Able to provide informed consent, or if the oncology physician determines the patient to not have decision-making capacity, a patient-designated health care proxy (or authorized representative per institutional policies) must sign consent by the baseline visit. If the participant is found to be impaired on the Blessed-Orientation Memory Concentration Test (BOMC) during screening; they must have a health care proxy or authorized representative to be eligible to enroll.

4.2.1g. Participant has adequate understanding of the English language because not all GA measures have been validated in other languages.

4.2.2. Exclusion Criteria for Patients

4.2.2a. Have surgery *planned* within 3 months of consent. Patients who have previously received surgery are eligible.

4.2.2b. Presence of symptomatic brain metastases at time of study consent process. Patients with history of treated brain metastases are eligible if they are not symptomatic at the time of study enrollment.

5. IDENTIFICATION, RECRUITMENT, AND CONSENT PROCEDURES

Patients will be recruited from the outpatient community oncology practices affiliated with the URCC NCORP Research Base network. The results of this study will be generalizable to the majority of older adults with cancer because it will include older cancer patients from diverse backgrounds and at varying health statuses who receive treatment in the community.

5.1. Study Participants

Study participants will be identified by their treating oncology physicians, who enroll in the study, the nurses that work with the enrolled oncology physicians and the clinical research associates (CRA). The

CRAs will work closely with the oncology physicians and nurses to identify those patients aged 70 and over with advanced cancer that are to begin a new cancer treatment regimen (see section 4.2.1c). The oncology physician then confirms if the patient is an eligible study candidate for all requirements other than GA impairment by completing the eligibility checklist with the CRA and signing it. The oncology physician or CRA will introduce the study to the eligible patients and/or their designated health care proxies or authorized representatives (per institutional policies), and will provide them with an IRB approved study brochure and consent to review. Adequate time will be provided to the patient and/or designated representative to read the consent. The CRA, the oncology physician, and the nurses are available to answer any questions the patient and/or authorized representative may have about any aspect of the study prior to consenting and throughout the entire study period. Patients and/or their designated authorized representative may choose to sign the informed consent document immediately on the day the study information is presented to them or they may choose to take the consent form home and discuss it with others; then if they want to participate in the study, they can provide signed consent forms the next time they meet with the CRA or oncology physician. Patients and/or their designated authorized representatives must sign consent prior to the oncology physician initiating the new cancer treatment regimen.

5.1.1. Informed consent will be obtained from the patient, unless they lack capacity to provide consent. If a patient lacks capacity, a health care proxy and/or authorized representative will be required to sign consent per institutional and/or local policies on consent for incapacitated/decisionally impaired patients. If the patient does not have an appointed health care proxy or authorized representative (per institutional policies) on or before the baseline visit, he/she will not be enrolled onto the study. All consent documents will be signed by the patient and/or designated health care proxy/authorized representative and maintained in the patient record with copies provided to the patient and/or designated health care proxy/authorized representative.

5.1.2. The screening measures will then be performed. Those with a diagnosis of dementia, as noted in their medical record or diagnosed by a physician, or who meet the cut-off score for impairment on the cognitive screen (score of 11 or more on Blessed Orientation-Memory-Concentration Scale (BOMC)) can be included if a designated health care proxy (or authorized representative per institutional policies) selected by the participant signs consent. The goal of the intervention is to improve outcomes of older cancer patients with all underlying health conditions including cognitive issues. Therefore we will include these patients and will conduct the assessments with assistance of the proxy.

5.1.3. Ethical standards for human subjects will be strictly followed in accordance with local policies and/or institutional review board requirements on the enrollment of adult decisionally incapacitated research participants and permission of authorized representatives (e.g., health care proxy). If the policy in 5.1.2 (as approved by the NCI and URCC IRB) violates local policies, the URCC NCORP Research Base should be notified if patients with cognitive impairment are not able to be enrolled by the site.

5.1.4. Current, state, federal, and institutional regulations concerning informed consent will be followed. Participation in this study is voluntary. Participants are free not to take part or to withdraw at any time, for whatever reason, without risking loss of present or future care they would otherwise expect to receive. Sections 11.4.1c and 14 include withdrawal procedures.

5.1.4a In the event that a participant does withdraw from the study, the information they have already provided will be kept in a confidential manner. Data will be used unless permission is revoked in writing and sent to their oncology physician or the URCC study team. Site oncology physicians should forward any such correspondence to the URCC Research Base.

5.2. Oncology Physician Recruitment

Because oncology physicians are being recruited and enrolled from sites across the country by the URCC Research Base, oncology physicians will read and agree to participate either via Research Electronic Data Capture (REDCap) or on paper if REDCap is not a feasible option. REDCap is a software toolset (see Section 16.3 for more information) for electronic collection and management of research and clinical trial data developed by Vanderbilt University. Oncology physicians will be enrolled prior to enrolling (screening registration) their first eligible patient. Staff from the URCC Research Base (including the PI) will be available to answer any questions the oncology physicians may have over the phone. Procedures for the oncology physicians are minimal risk and involve completing surveys which will be de-identified and identifying patients for the study.

Oncology physicians will provide baseline demographic information, fill out a baseline survey that evaluates preferences for decision-making, and help identify their own patients who may be eligible for the study. The baseline survey can be completed online via REDCap or on paper if REDCap is not feasible. REDCap will securely store the oncology physician's email address for surveys. In addition to the email address, the only personal identifying information the oncology physicians will provide will be their name, age, ethnicity, and the name of the clinic where they work. The oncology physician will be assigned an ID number, which will be used to link their surveys with those of their patients enrolled in the study.

The participation of oncology physicians in the research study meets criteria for "waiver of documentation of consent" because the research involves no more than minimal risk to the oncology physicians and there are no procedures for the oncology physicians that would normally require written consent outside of the research context. Therefore URCC Research Base is responsible for tracking and collecting physician agreement to participate in the study. Hence, the NCORP site staff do not need to consent their participating Physician.

6. REGISTRATION

6.1. Prior to entering participants, either oncology physicians or patients, on this protocol, the following must be on file at the URCC NCORP Research Base:

- **6.1.1.** Documentation of IRB approval in the form of an HHS Protection of Human Subjects Assurance Identification/IRB Certification/Declaration of Exemption (formerly Form 310), CTSU approval form or signed letter from the IRB.
- **6.1.2.** A copy of the institution's IRB-approved informed consent document.
- **6.1.3.** Written justification for any substantive modifications made to the informed consent concerning information on risks or alternative procedures. Written justification should be provided also regarding changes related to the inclusion or consent of decisionally incapacitated participants (see sections 5.1.2 and 5.1.3).

These documents are submitted to:

Cathleen_Lesniewski@urmc.rochester.edu

OR

Ms. Cathy Lesniewski
URCC NCORP Research Base
Saunders Research Building
265 Crittenden Blvd
CU 420658
Rochester, NY 14642

6.2. Registration Requirements

6.2.1. Timing of Registration: See section 11, Outline of Study-Specific Procedures.

6.2.2. Screening Registration

6.2.2a. Go to the URCC NCORP Research Base website at URCC-NCORP.org and enter the information outlined below.

- If you experience difficulties you may call 585-275-1364 at the URCC NCORP Research Base to verbally give the URCC registrar the information below.
- The following information will be requested:
  - NCORP Affiliate Name
  - URCC Protocol
  - Most recent IRB approval date (either initial or annual, NOT the date of approval of an amendment)
  - NCORP Practice Site
  - Name and telephone number of person registering study participant
  - Confirmation that consent form has been signed and by whom
  - Confirmation of participant screening ID if participating in another URCC study
  - Participant's Physician Name (confirms that oncology physician has been enrolled)
  - Participant's identification
    - First and last initials

6.2.3. Baseline Registration

6.2.3a. Go to the URCC NCORP Research Base website at URCC-NCORP.org and enter the information outlined below.

- Baseline registration can be completed up to 2 work days prior to the scheduled baseline visit.
- The geriatric assessment and baseline registration should be completed within 4 weeks after the screening visit.
- If you experience difficulties you may call 585-275-1364 at the URCC NCORP Research Base to verbally give the URCC registrar the information below.
- The following information will be requested:
  - NCORP Affiliate Name
  - URCC Protocol
  - Most recent IRB approval date (either initial or annual, NOT the date of approval of an amendment)
  - NCORP Practice Site
  - Name and telephone number of person registering study participant
  - Confirmation that all eligibility requirements have been met
  - Confirmation that consent form has been signed and by whom
  - Participant's screening ID
  - Participant's Physician Name (confirms that oncology physician has been enrolled)
  - Participant's identification
    - First and last initials
    - Birth date (MM/DD/YYYY)
    - Gender
    - Race/Ethnicity
    - Five-digit zip code
    - Payment code

- Confirmation of participant assessment scores for the Blessed-Orientation Memory Concentration Test (BOMC) and the Geriatric Depression Scale (GDS)
- Planned chemotherapy or other approved agents with similar toxicity prevalence
- Date of Baseline Visit

- **An email confirmation of registration will be forwarded by the URCC Research Base.**

6.3. A total enrollment of 700 participants is planned.

6.4. This protocol is open only to affiliates of the URCC NCORP Research Base who provide written documentation of IRB approval. There will be no accrual at the URCC NCORP Research Base itself.

7. OUTCOMES

7.1. Clinical Outcomes

7.1.1. Toxicity. Toxicity will be captured in a standardized manner using the NCI Common Terminology Criteria for Adverse Events (V4.0). The NCI's Common Terminology Criteria for Adverse Events (CTCAE; http://ctep.cancer.gov/reporting/ctc.html) is a longstanding empirically developed "dictionary" or lexicon, designed for use in clinical trials to aid clinicians in detecting and documenting an array of adverse events (AEs) commonly encountered in oncology. An AE is any unfavorable sign (including an abnormal laboratory finding), symptom, or disease temporally associated with the use of a medical treatment or intervention that may or may *not* be considered related to the medical treatment or intervention under investigation. The AE may be either unexpected or expected.

An AE is a term that is a unique representation of a specific event used for medical documentation and scientific analyses of treatment efficacy and tolerability. Each AE is typically graded on a scale of 1 (mild) to 5 (death related to AE), though a grade 5 is not relevant for some AEs, such as hair loss or skin itching. The reporting requirements for AEs are generally protocol-specific and may be divided into two types. The first is the protocol-specific AEs to be addressed at designated evaluation intervals. The second is the pertinent positive clinical signs, symptoms, and laboratory results obtained as part of routine care of patients. The CTCAE is maintained by the NCI's Cancer Therapy Evaluation Program (CTEP). The CTCAE is currently in its fourth version.

Ideally, the CRA will be present at each scheduled visit where toxicities are captured and graded by the oncology team. In the event that the CRA is unable to be present at the visit, s/he will need to follow-up with the oncology team to verify the toxicities. Treatment dosing administration as well as grade 2-5 treatment toxicities will be captured for 3 months after study enrollment. Toxicities will be captured for all treatments provided within the first 3 months. Grade 2-5 treatment toxicities and treatment dosing administration will be captured between 3-6 months only for those participants who remain on any of the same treatment drugs that they were receiving at study initiation (including for those who treatment dosing was modified or delayed).

Per participant, the CRA will complete the forms capturing dosing administration and grade 2-5 toxicities for each treatment cycle to summarize the above information, which will be sent to the Research Base after study visits. Treatment dosing administration and toxicities will be captured either by standard forms or electronically through REDCap. REDCap (see section 16.3) will capture exactly the same information as the forms and will be available as a data entry option for capture of treatment and toxicity information.

The medical record will also be reviewed in order to capture each clinical encounter (scheduled or emergency visits). This will include a review of the clinic notes, emergency room visits, and

hospitalizations (i.e., emergency room and hospitalization discharge summaries). If the participant seeks emergency care outside of the primary institution, the participant's permission will be obtained to review these outside records. Details regarding the overall category of toxicity (hematologic or non-hematologic), specific type of toxicity, and the rationale for the toxicity grade will be captured. Dose reductions, dose delays or discontinuation of the treatment course will also be captured, as well as the cause (i.e. relationship to toxicity).

7.1.2. Cancer Treatment Decisions. The NCCN guidelines[105] will be utilized to capture the standard dosing of treatment regimens. The planned regimen (individual drugs, doses, and schedule) will be captured at the beginning of the study from the primary oncology team. The cumulative dosages per unit time of the individual drugs in the regimen will be calculated: (total mg of drug in all cycles/m^2 body surface area)/(total days of therapy/7). The denominator is based on total days on treatment (from day 1 of cycle 1 through 1 cycle length after the date of the last treatment), reflecting all dose delays. The relative dose intensity (RDI) is calculated as the ratio of the amount actually delivered to the amount intended based on standard guidelines. The actual dose delivered (in the numerator of RDI) will account for dose reductions. The RDI is calculated for each drug in a multidrug regimen, which are averaged to derive the average RDI. The doses will be collected in the forms completed by the CRAs at the sites. All calculations will be performed at the Research Base.

7.1.3. Survival. We will capture survival through the participant's medical record and verification with the primary team. We will follow participants for survival for 12 months after enrollment. We will obtain the date, location of death, and cause of death. If a site becomes aware that a study participant is now deceased, they should complete the Survival Status form which is available on the URCC NCORP website. Otherwise sites will be contacted approximately 1 year after each participant was enrolled to assess survival and asked to complete this form. We will also verify information with Medicare claims data if the participant provided permission to do this through initial consent (see Section 7.2.3).

7.2. Data Sources

7.2.1. Participant Surveys. Participants will complete surveys prior to the start of treatment and at 4-6 weeks, 3 months, and 6 months. We are sensitive to respondent burden and have minimized the number of items to be completed in a single sitting. All surveys have been utilized in our pilot work with older patients with cancer and other age-related health conditions. In a recent study, 98% of patients with non-small cell lung cancer with a median survival of 9 months completed a baseline battery similar in length, and 70% and 64% of those who were still alive were able to complete assessments at 3 and 6 months, respectively.[106,107] As is often true for patients with advanced disease, missing data were *not* random; sicker patients tended not to complete surveys. We have included approaches to missing data in section 11.4.1c. and in the statistical section of the protocol. Non-completion of surveys due to illness or fatigue should not be considered a patient withdrawal. Every effort should be made to collect participant survey information in person and objective GA information. If survey and GA information cannot be collected (due to participant illness and fatigue), CRA forms regarding treatment and toxicity information should still be completed at the appropriate time points. In addition, clinic notes, treatment records, and emergency room/hospitalization discharge summaries should also be provided. Hospitalization discharge summaries are not required for participants in hospice or end of life care.

7.2.2. Oncology Physician Surveys. Oncology physicians will complete a baseline survey prior to or when their first patient consents to the study and a brief follow-up survey at the end of the study. After the baseline visit, oncology physicians will be asked about potentially important covariates or moderators, including disease and treatment characteristics. They will also complete a brief survey about decision-making after each follow-up visit. When an oncology physician's study participation

is near completion (e.g., all their study participants are off study, they are moving or leaving the practice) the oncology physician will complete the Final Physician Survey found on the NCORP website with a separate version for the control and the intervention sites.

7.2.3. Chart Abstraction and Claims. Clinic notes, treatment records, and emergency room/hospitalization discharge summaries will be requested to validate treatment dosing and toxicity information. Hospitalization discharge summaries are not required for participants in hospice or end of life care. If there is missing information or conflicting medical information from the surveys, we will obtain additional medical records in order to verify information about disease location, pathology, stage, and metastases from charts. We will request information from the CRA on the final treatment recommendations made and implemented. In order to assess health care utilization (e.g., adverse events such as hospitalizations) for future work on examining cost-effectiveness of the intervention, permission to obtain Medicare claims in the future will be asked on the consent form. Claims will not be obtained for any individual participant until the participant is off study. Claims information will be collected for all participants that agreed per the consent, including those who have completed the study or have withdrawn from the study. All consent and research procedures for obtaining Medicare claims will be followed: http://www.cms.gov/Research-Statistics-Data-and-Systems/Computer-Data-and-Systems/Privacy/Researchers.html

Permission to obtain claims is voluntary. Patients will be able to decline this procedure at the time of consent. Declining consent for obtaining claims from Medicare for future research to examine cost-effectiveness, quality of care, and health care utilization does not preclude patients from participating in the rest of the study.

8. MEASURES TO BE COLLECTED

An overview of measures is provided here. A detailed description of each measure including a summary table is provided in **Appendix A**.

**Table 1: Participant and CRA Measures:**
Demographics
Medical Characteristics and Treatment
Labs/Lab Form
Com-meds/Polypharmacy
Geriatric Assessment
- Assessments by CRA
- GA Participant Packet
Decision-Making Preferences
PRO-CTCAE
Clinician-rated CTCAE (Primary Outcome)

We have piloted all measures. In total, geriatric assessment measures that are filled out by the participant require approximately 20 minutes of time. The additional measures (e.g., PRO-CTCAE) captured at baseline require an additional 15 minutes of time. We have incorporated flexibility with timing in order to reduce participant burden. The follow-up surveys require about 30 minutes of time in total.

Participants may complete the first geriatric assessment in clinic at time of consent or in clinic before the next visit with the oncology physician. They may choose to complete measures at home in between visits. The geriatric oncology clinic at the University of Rochester routinely captures these measures as part of clinical care.

The assessments performed by the CRA and associated surveys take about 30 minutes of time in total (including physical performance and cognitive tests). *Any person at the practice site can be trained by Research Base staff to do the GA. The GA does not need to be performed by the oncology physician.*

The oncology physician assessments will be done either on paper or through REDCap, whichever the oncology physician prefers. The baseline and end of study assessments take no longer than 10 minutes and after the initial participant visit, the decision-making form (to assess factors that influenced decisions) takes only a few minutes to complete.

Components of Geriatric Assessment (Participant)
Assessment tools comprising the comprehensive GA are listed in **Table 2.** The various assessment tools were selected based upon extensive data in the geriatric literature demonstrating predictive value as well as feasibility data in multiple studies of elderly patients with cancer. Other than the cognitive and physical performance measures, the assessments are self-administered. Participants who cannot complete the assessment on their own will receive assistance from study personnel or a caregiver. The GA is performed before baseline registration. Follow-up GA measures are collected at 4-6 weeks, 3 months, and 6 months.

Table 2: Components of the Comprehensive Geriatric Assessment

DOMAIN	TOOL	SCORE SIGNIFYING IMPAIRMENT
**Physical function**	➢ Activities of Daily Living<br>➢ Instrumental ADLs<br>➢ Fall History<br>➢ OARS Physical Health	➢ Any ADL deficit<br>➢ Any IADL deficit<br>➢ Any history of falls<br>➢ A lot of difficulty with any task
**Objective physical performance**	➢ Short Physical Performance Battery<br>➢ Timed "Up and Go"***	➢ ≤ 9 points<br>➢ >13.5 seconds
**Comorbidity**	➢ OARS Comorbidity***	➢ Participant answered "yes" to ≥3 chronic illnesses<br>➢ One illness interferes "a great deal" with QoL
**Nutrition**	➢ Body Mass Index<br>➢ Mini Nutritional Status***<br>➢ Weight loss***	➢ <21 kg/m^2<br>➢ ≤ 11 points<br>➢ >10% from baseline weight
**Social support**	➢ OARS Medical Social Support***	➢ Participant answers one of the social support questions indicating less than adequate support for care
**Polypharmacy**	➢ Polypharmacy<br>➢ Lab***	➢ ≥5 regularly scheduled prescription medications OR<br>➢ Any high-risk medication OR<br>➢ Creatinine clearance <60 ml/min
**Psychological**	➢ GAD-7 ***<br>➢ Geriatric Depression Scale	➢ ≥ 10 points<br>➢ ≥5 points
**Cognition**	➢ Blessed OMC***<br>➢ Mini-Cog	➢ >10<br>➢ 0 words recalled OR 1-2 words recalled + abnormal clock drawing test

*****Captured before baseline registration**

Abbreviations: ADL (Activities of Daily Living); Blessed OMC (Blessed-Orientation Memory Concentration Test); GAD (Generalized Anxiety Disorder 7-Item Scale); GDS (Geriatric Depression Scale); IADL (Instrumental Activities of Daily Living); QoL (Quality of Life).

8.2. Other Clinical Measures (Participant)

8.2.1. Sociodemographics.*** Participant age, race and ethnicity, gender, highest level of education achieved, employment status, marital status, and presence of a living companion will be captured. We will also assess understanding of disease.

8.2.2. Tumor and Treatment Characteristics. The tumor stage, previous surgery or radiation, previous cancer treatments, current cancer treatment plan including chemotherapy (type, dosing, and schedule), monoclonal antibody treatment, targeted agents, and supportive care medications will be captured by the CRA.

8.2.3. PRO-CTCAE. *(from*: http://outcomes.cancer.gov/tools/pro-ctcae_fact_sheet.pdf) There is growing awareness that collecting symptom data directly from patients using patient reported outcome (PRO) tools can improve the accuracy and efficiency of symptomatic AE data collection. This is based on findings from multiple studies demonstrating that physicians and nurses underestimate symptom onset, frequency, and severity in comparison with patient ratings. For example, in a study of men with prostate cancer enrolled in a Phase II clinical trial, physician

reporting was neither sensitive nor specific in detecting common chemotherapy symptomatic adverse effects.[108]

In the field of pain management, it has long been recognized that only the patient can accurately report the onset, severity and duration of pain and its impact upon function. This principle extends to other symptoms, such as fatigue, erectile dysfunction, and xerostomia (dry mouth), which can be found in the CTCAE. The other advantages of a PRO complement to the CTCAE are discussed in an article by Trotti et al.[109]

The NCI's Patient-Reported Outcomes version of the Common Terminology Criteria for Adverse Events (PRO-CTCAE) system provides a platform to collect patient reports of symptoms they are experiencing while undergoing treatment, for the purpose of enhancing adverse event (AE) reporting http://outcomes.cancer.gov/tools/pro-ctcae.html). To date, 81 symptoms of the CTCAE (version 4) have been identified as amenable to patient reporting. These symptoms have been converted to patient terms (e.g., CTCAE term "myalgia" converted to "aching muscles").

For purposes of this project, core items will be assessed along with items related to geriatric functioning. Participants will complete these surveys at baseline, 4-6 week, 3 month, and 6 month time points.

8.3 Decision-Making Assessments (Participant and Oncology Physician)

8.3.1. Participant Assessments. We will collect measures to assess the processes of decision-making for treatment. The measures chosen for this study are validated tools designed to measure how participants' approach the decision-making process. Validated tools will measure patient-centered autonomy-supportive oncology physician behaviors such as whether the participant feels that the oncology physician understands his/her perspective, and provides choices and options.

8.3.2. Oncology Physician Assessments. We will collect information on demographics and decision-making preferences. Oncology physicians will be presented with a clinical scenario of elderly cancer patients with a variety of geriatric-related impairments (i.e., physical frailty, cognitive impairment). A series of questions will follow each vignette inquiring about the likelihood of the oncology physician to offer chemotherapy in the scenario. After the study is over, physicians will be asked to complete a brief follow-up survey. After the baseline clinical encounter, oncology physicians will complete a short (<10 question) survey follow-up requesting information on the treatment plan for the participant and factors that influenced how the decision was made.

9. NCORP PRACTICE SITE RANDOMIZATION

A practice site is defined as any practice location within an overarching NCORP designation where oncology physicians and study staff work independently, i.e., do not cross over into another site. Practice sites will be randomized to one of the two study arms by means of a computer–generated randomization table. Past accrual to URCC studies will be used to stratify each practice site as a large accruing (20 or more accruals/year) site or a small accruing practice site in order to assure balance in the randomization. The randomization process will be determined using R software provided by Dr. Charles Heckler, the lead biostatistician of the URCC NCORP Research Base.

10. INTERVENTION OVERVIEW FOR PHYSICIANS

Practice sites will be notified if they are in the intervention arm by URCC Research Base staff. At the sites randomized to the intervention arm, oncology teams will implement the intervention. The oncology physician is required to review the GA information and decide upon recommendations. He/she can designate an advanced practice practitioner (APP) (e.g., nurse practitioner (NP) or physician assistant (PA))or an oncology fellow to assist with intervention procedures. The URCC NCORP Research Base must be informed if an APP or a fellow is participating prior to any study procedures being implemented. Designated APPs and fellows, if at an intervention site, must complete intervention training procedures.

Sites will be asked by the Research Base staff if there are members on the oncology team who may conduct the baseline visit at the time of IRB approval or after a physician is recruited. These members will be contacted and trained appropriately for their group assignment (i.e. control, intervention).

Prior to implementing the intervention, oncology physicians and designees will receive training on how to best utilize GA information in clinical practice for older adults with cancer. The training session provides a brief overview of the intervention information and can be completed through a web conference, telephone call, or as a paper "sign-off" documenting review of the materials. The training materials were developed with resources from Drs. Mohile's and Hurria's geriatric oncology lectures.

The CRAs and/or research nurses at sites randomized to the intervention arm will be trained to utilize the *mycarg.org website* to derive a summary of GA scores and a list of targeted GA-driven recommendations. This information will be printed by the site CRAs for the oncology physician who will "sign off" that he/she has received and reviewed the information.

Study or clinical staff can assist the oncology physician in checking which recommendations were considered and facilitating implementation of GA-driven recommendations. The oncology physician can identify which of the recommendations he/she thinks are appropriate and high priority for the participant. They do not all need to be implemented at the time of the visit but implementation should occur before the 4 week visit.

Selection of GA recommendations could occur prior to the baseline visit or during the baseline visit in conjunction with the participant and/or caregiver. It is strongly preferred that the oncology physician conduct the baseline study visit. The baseline study visit can be conducted by the physician in conjunction with the APP or fellow. If the oncology physician is not available prior to the start of the new treatment regimen, an APP or oncology fellow may conduct the baseline study visit. The APP and/or oncology fellow must have completed intervention training prior to the baseline study visit. If an APP or oncology fellow is conducting the baseline visit, the oncology physician should still determine the recommendations and they will need to be approved or "signed off" by the oncology physician. It is required that discussion occurs either in person or by telephone between the oncology physician and APP or oncology fellow regarding the GA information and recommendations prior to the baseline visit. The oncology physician must still complete the other physician forms. The baseline surveys will capture which member of the oncology team conducted the baseline visit.

Two copies of the summary and checklists should be made: one should be provided to the participant/caregiver and one should be retained in the study chart. Participants and caregivers should be provided with a copy of the GA summary and GA-driven recommendation forms prior to the baseline visit. The original forms should be submitted to the URCC Research Base.

11. OUTLINE OF STUDY-SPECIFIC PROCEDURES

Usual Care Site (Arm 2) | **Intervention Site (Arm 1)**

Oncology Physician Enrollment – Baseline survey on REDCAP
Subject Identification Process

Screening: Visit 0

- Consent patient; complete screening registration.
- If time permits, administer GA measures (cognitive, physical performance, and nutrition) to participant.
- Participant complete screening packet during visit or take the screening packet & baseline packet home with them to complete prior to the baseline visit.
- CRA completes CRA screening study forms and confirms planned treatment regimen is an approved one by checking on the URCC Research Base list
- Confirm participant plans to start a new regimen and will bring completed packet to next visit or schedule additional time if needed to complete at visit.

Score GA & Complete Baseline Registration

- When participant and CRA screening forms complete, score each GA measure as per training procedures (at screening or beginning of baseline visit prior to study visit with oncology team).
- Complete baseline registration for participant if participant has 1 or more domains that meet cut-off score for impairment (other than polypharmacy).

Baseline: Visit 1 – Usual Care

- Inform oncology physician if depression (GDS) or cognition (BOMC) assessments score ≥ 11.

Baseline: Visit 1 – Intervention

- CRA enter GA Score on MYCARG.ORG
- Print and provide GA summary and recommendations forms to oncology team member*
- Information on cognitive impairment or depression is included in summary.

- Study visit with oncology team* occurs
- Oncology physician completes forms about participant
- Submit all materials to URCC within 7 days.

All Follow-up Visits: Visit 2, Visit 3, Visit 4
(Visits are 4 to 6 weeks, 3 months, and 6 months from Baseline)

Before Visit: Confirm participant will bring completed packets to study visit.
During Visit: Administer cognitive and physical performance measures and complete CRA study forms. **Intervention Arm ONLY: CRA complete GA-driven recommendation follow-up forms at visit 2 (4-6wks).* Inform oncology physician if depression (GDS) assessment score ≥ 11.
After Visit: Submit all forms to URCC Research Base within 7 days.

*Oncology team member can include oncology physician, APP, or oncology fellow. If an APP or oncology fellow is conducting the baseline visit, the recommendations still need to be approved or "signed off" by the oncology physician. It is required that discussion occur between the oncology physician and APP or oncology fellow regarding the GA information and recommendations prior to the baseline visit. The oncology physician must still complete the other physician forms.

11.1. Procedures Prior to Screening Visit

11.1.1. Oncology Physician Enrollment and Participation

11.1.1a. If an oncology physician is interested in the study and meets the inclusion/exclusion criteria, he/she will agree to participate on paper or through REDCap (sent by the URCC Research Base).

11.1.1b The oncology physician will complete a baseline survey on demographics and treatment preferences either on paper or through REDCap.

11.1.1c Ideally oncology physicians are enrolled as soon as possible. However, an oncology physician can be enrolled after his/her patient is identified for the study or prior to screening registration for the first identified patient.

11.1.2. Participant Identification Process

11.1.2a. Participants: Once a site has IRB approval, the site study staff can start screening for participants according to the inclusion/exclusion criteria for patients (section 4.2). Only patients from enrolled physicians can be registered.

11.1.2b. Screening Log: A screening log will be kept at each participating site, where *all* patients *approached for the study* will be entered by site study staff. The screening log can be completed on paper or through REDCap.

11.2. Screening: VISIT 0

11.2.1. Participant Recruitment & Consent Process

11.2.1a. Participants

- The site study staff will notify the patient's oncology physician when a patient is identified as a possible candidate for the study. Site study staff should screen for patients who may fit eligibility criteria for all requirements other than GA impairment. It is anticipated that some of the patients who are eligible will be new patients for the oncology physician. Site study staff should confirm that planned treatment includes either a chemotherapy agent or another agents approved by the URCC NCORP Research Base. Approved agents are available on a list on the URCC NCORP Research Base website with study-related materials. If the agent or regimen is not on the list, contact URCC NCORP Research Base for approval. The Research Base will review toxicity information and if approved, will add drugs to the list in an ongoing fashion (see section 4.2.1c).
- The oncology physician and/or CRA should mention the study to the patient and give out recruitment materials.
- If the patient is interested, the oncology physician and/or site study staff will explain the study and once all aspects of the study have been discussed to the patient's satisfaction, the voluntary written informed consent procedures will be completed with the patient if they choose to enroll in the study.
- The site study staff can schedule a separate visit with the patient to go over consent and initiate study procedures if more time is needed.

11.2.2. Participant Assessment Process

11.2.2a. Once a participant has consented, screening and baseline assessments/procedures need to

occur prior to starting the new regimen. Site study staff can schedule a separate visit with the participant to complete study procedures if more time is needed.

11.2.2b. The procedures for screening will consist of the steps below:

- Confirm that the participant's oncology physician has been enrolled onto the study and if not, follow procedures for enrolling oncology physicians.
- Participants meeting eligibility criteria are initially registered with a screening registration *after* signing consent.
- Administer cognitive, physical, and nutritional assessments to the participant.
  - These assessments can be administered right after consent, at a separate visit scheduled by site study staff, or just prior to the baseline study visit. Allow 45 minutes to perform assessments if done just prior to the baseline study visit.
  - Anyone at the practice site can administer cognitive and physical assessments to the participant as long as they have participated in the required GA training administered by the URCC Research Base. *CRAs, clinical nurses, and technicians are all eligible to participate in training and can complete GA study procedures.*
- Participant will complete all screening surveys.
  - Ideally the participant will complete either at the time of consent or the screening and baseline packets can be taken home and completed before the baseline study visit. If the participant needs assistance from site study staff to complete surveys, allow adequate time. Approximately 60 minutes is recommended.
- Abstract required medical information from chart.

11.2.2c. The GA must be completed within 4 weeks prior to the baseline study visit with the oncology team. The baseline study visit with the oncology team should be scheduled prior to starting the new treatment regimen. Treatment should begin within 4 weeks after the baseline study visit.

11.3. Baseline: VISIT 1

11.3.1. Participant Assessment Process

11.3.1.a. BEFORE BASELINE VISIT:
Site study staff will telephone the participant to:

- Confirm that participant will be starting a new treatment regimen. If a participant decides not to proceed with the new regimen before the baseline registration is performed, registration should not be completed and the participant should be considered a screen failure.
- Confirm scheduled visit with the oncology team.
- Confirm that the participant completed the screening surveys before the baseline study visit and remind them to bring the surveys in with them.
  - Schedule the participant to come in to meet with site study staff at least 45 minutes prior to the baseline study visit in order to complete study procedures. Allow for more time if participant communicates that surveys have not been completed. Only participants that complete GA procedures can be registered for the study.

11.3.1.b. AT THE BASELINE VISIT:

- If screening surveys have not been completed prior to the participant coming in, complete them at this visit.
- If not complete, staff should administer cognitive, physical performance, and nutrition assessment.

- If not complete, staff should complete CRA study forms.
- If not complete, baseline surveys should be done. The baseline surveys do not need to be completed prior to baseline registration, but should be completed on the day of the baseline visit. For example, participants can complete baseline surveys while receiving cancer treatment that same day.
- Score each GA measure as taught in training procedures. If assistance is needed for scoring, contact the URCC NCORP Research Base.
- Participants that have at least one abnormal GA score other than polypharmacy can move forward with the study.
- After the GA and the above steps are complete, the baseline registration procedures can be completed for the participant (refer to section 6. Registration).
    - Registration must occur prior to baseline study visit with the oncology team and prior to starting the new treatment regimen.
    - Once the participant is registered, if the new treatment regimen does not get initiated, complete the Cancer Treatment Status form. Participants should continue to be followed and remain on study.
- Prior to the study visit with the oncology team, if depression (GDS) or cognition (BOMC) assessments score ≥ 11, inform participant's oncology physician as follows:
    - Usual Care arm -- inform oncology physician with template as per training.
    - Intervention arm -- information on cognitive impairment or depression is included in summary (see section 11.3.2, *Intervention Procedures*).

11.3.2. Intervention Procedures (<u>Only for sites randomized to the intervention arm</u>)

11.3.2.a. AT THE BASELINE VISIT:

- After the participant has been registered for the study, enter the GA scores into mycarg.org website or if no internet is available, contact the URCC NCORP Research Base Program Manager to assist.
- After entering the GA scores, print the built pdf packet specific to that participant.
- Present GA summary form and recommendation forms to oncology team to review just prior to baseline study visit. ***The study visit must occur prior to starting the new regimen***. Study staff can assist in the completion of the forms. Study and clinical staff can assist in implementing GA-driven recommendations after the physician approves them.
    - Information on cognitive impairment or depression is included in the summary.
    - The oncology physician MUST review and sign the summary form and recommendations forms. Another team member (APP or oncology fellow) can conduct the baseline study visit only if the physician is unavailable. If this is the case, the GA summary information and recommendations must be reviewed or discussed with the oncology physician prior to the baseline study visit.
    - GA recommendations that are planned by the oncology physician for the participant should be check-marked by the end of the visit.
- Two copies of the GA summary and GA recommendations forms should be made: one should be provided to the participant and one should be retained in the study chart.
- The original forms should be sent to the URCC Research Base.

11.3.4. Oncology Physician Assessment for Participant: After the baseline visit, oncology physicians will complete a brief survey (on paper) to capture factors that influenced the decision-making process for treatment. Oncology physicians must complete this survey even if another member of the oncology team conducted the baseline study visit.

11.3.5. Submitting Materials to the URCC Research Base:

11.3.5a. After the baseline visit, site study staff should ensure the forms below are completed and submit them to the Research Base:

- All oncology physician and participant assessments from above
- All CRA study forms
- Clinic note from visits
- Cancer treatment plan
- Intervention summary and recommendation forms (if applicable)

11.4. Follow-up Visits:

11.4.1. Visits will occur 4-6 weeks after the baseline visit, 10-14 weeks after the baseline visit (3 months), and 20-26 weeks after the baseline visit (6 months).

11.4.1a. BEFORE EACH FOLLOW-UP VISIT:
Study staff will telephone the participant to:

- Confirm scheduled visit with the oncology team. The visit does not need to be scheduled with the oncology physician. The participant can choose to visit with study staff to complete procedures on a day other than a scheduled visit with their oncology team.
- Confirm that the participant completed the surveys.
  - Surveys must be completed during the appropriate window for each time point. Site study staff can either mail the follow-up surveys OR at the end of one visit, they can provide the next round of surveys to the participants to take home with them for the next follow-up visit.
  - Ideally participants will complete the survey packet at home and bring to site study staff on the day of a routinely scheduled visit for the oncology physician or designee (e.g., nurse practitioner) visit or during cancer treatment infusions.
  - If the participant needs assistance from site study staff to complete surveys on-site, allow adequate time (45-60 minutes).
  - If participants cannot complete packet at home:
    - Participant can come in prior to office visit to complete surveys.
    - Participant can complete surveys during cancer treatment infusions.
    - If needed, the participant can meet with the site study staff at an additional time to complete the follow-up surveys. The participants do not need to meet with the oncology physician to complete the surveys.

11.4.1b. AT SCHEDULED FOLLOW-UP VISITS:

- *Participant:* Bring in completed surveys in the follow-up packet or complete at visit.
- *At each visit: Staff* will review participant surveys for completion and administer the following assessments to the participant:
  - Cognitive and Physical Performance
  - If depression (GDS) assessment score ≥ 11, both usual care arm and intervention arm inform participant's oncology physician with template as per training.
  - Complete all CRA follow-up study forms including treatment and toxicity forms. Treatment and toxicity information should be captured consecutively.
- *At the 4-6 week time point:* CRAs at practice sites randomized to the **intervention arm** will complete the GA-Recommendation Follow-up forms by comparing them to the GA-Recommendation Baseline forms, clinic notes, and conferring with the oncology team if needed to verify which recommendations were implemented.
- If the participant discontinues cancer treatment for any reason, a Cancer Treatment Status

form should be completed and submitted to the URCC NCORP Research Base and the participant should continue to be followed on study.

- Site study staff will submit the following to the URCC Research Base:
    - All oncology physician and participant surveys
    - Clinic note from visits
    - Chemotherapy and other cancer treatment records
    - Emergency room or hospitalization discharge summaries
    - All CRA, participant, and physician study forms

Other medical records will be requested if there are discrepancies or missing information in key data.

11.4.1c. DEFINITIONS AND PROCEDURES RELATED TO ACTIVE WITH MISSING DATA, LOST TO FOLLOW-UP, AND WITHDRAWAL

Many in this study's participant population are expected to become more ill as the study progresses due to the nature of their underlying cancer diagnosis. It is understood participants may not be well enough to complete all study assessments or visits. Therefore, the following definitions are provided to account for this when capturing patient status outcomes in this population.

Once a participant has completed the baseline registration, participants are considered **active participants with missing survey data** if they decline to complete surveys or CRA administered GA measures or both (e.g., too ill, too tired) or they are unable to come to study visits due to being too ill or entering hospice. They will be considered lost to follow up if they are unable to be contacted by calls and emails.

Participants who discontinue treatment but are still being followed by the site should still be requested to complete study procedures (surveys and CRA administered GA measures). If they decline to complete surveys or CRA administered GA measures, these participants will also be considered active participants with missing survey data.

In all of the above scenarios, CRA measures collecting data from the medical record (clinic notes, chemotherapy flow-sheets, labs) should continue until the 3 month time point for all participants receiving any treatment. At the 6 month time point, treatment and toxicity data will be collected for participants who are continuing on any of the original treatment drugs even if dose adjustments have been made.

**Collected in addition to right shaded panel if participant declines to complete surveys but still agrees to CRA-administered GAs**		**Collected if participant declines or cannot complete surveys AND CRA-administered GAs**		
• Nutritional Status and MNA	• BOMC	• Polypharmacy Log	• Cancer Treatment Dosage Form	• Cancer Treatment Status Follow-up Form (if applicable)
• Mini Cog	• Timed "Up and Go"	• Physician Rated KPS	• Toxicity Outcomes	• GA Recommendation Forms at 4-6 week visit
• Short Physical Performance Battery		• Decision Regret Follow-up /Physician		

The primary aim of this study requires the review of treatment toxicity through patient report and/or CRA review of the medical record (in collaboration with the oncology team). Assessment of treatment toxicity is a routine part of clinical care. As noted above, patients can partially or completely discontinue their own participation in study activities at any time without actively (verbally) withdrawing from the study (i.e., active participant with missing data). A withdrawal from the study is defined as a patient who verbally states that they no longer wish to be participating in, contacted or associated with this study. Only a patient can withdraw themselves from the study. If the patient does not have cognitive capacity, their proxy or authorized representative can withdraw a patient from the study. CRAs cannot withdraw a patient from study.

Because of the fragile patient population being studied, the patient should not be automatically assumed to be withdrawn from the study if they are too ill to complete surveys or if they decline to complete surveys or GA procedures at one or more visits or go into hospice. The CRA should continue to obtain medical chart data, as it relates to subject outcomes, treatment and toxicity outcomes (i.e., Cancer Treatment Dosage Form, Toxicity Outcome Forms, Cancer Treatment Status Follow-up Forms) for both active with missing data and withdrawal patients unless the patient provides in writing that they no longer wish this data to be collected. This should be provided to the UR NCORP Research Base. These procedures do not require patient participation. Research Base staff will also obtain Medicare data if agreed to in the patient consent.

All data will be kept in a confidential manner. All data will be used unless permission is revoked in writing and forwarded to the URCC NCORP Research Base. Site staff should forward any such correspondence to the URCC NCORP Research Base.

11.5. Survival Status will be recorded at approximately one year from date of participant baseline registration on the Survival Status form.

12. REIMBURSEMENT

In order to improve study retention and compliance, we will compensate participants for their participation (i.e., time and travel). Assessments are paid at $15.00 each and payments will be provided after each time point starting with the baseline visit. Participants can receive up to $60 if all four time point assessments are completed.

13. ADVERSE EVENT REPORTING

13.1. Risks from participating in this research are psychological distress from completing the questionnaires and the cognitive testing.

13.2. ***ADVERSE EVENTS RELATED TO STUDY PROCEDURES AND NOT TO ROUTINE ONCOLOGY TREATMENT AND CARE*** will be reported using the URCC Adverse Event form. This form can be found on the URCC NCORP Research Base website.

13.3. Adverse Events will be reported in accordance with the following guidelines:

	Grade 1	Grade 2			Grade 3				Grade 4		Grade 5	
	Unexpected and Expected	Unexpected		Expected	Unexpected		Expected		Unexpected	Expected	Unexpected	Expected
		with hospital-ization	without hospital-ization		with hospital-ization	without hospital-ization	With hospital-ization	without hospital-ization				
Unrelated Unlikely	Not Required	Not Required	Not Required	Not Required	Not Required	Not Required	Not Required	Not Required	10 Calendar Days	Not Required	10 Calendar Days	10 Calendar Days
Possible Probable Definite	Not Required	10 Calendar Days	Not Required	Not Required	10 Calendar Days	10 Calendar Days	Not Required	Not Required	24-Hour; 5 Calendar Days	10 Calendar Days	24-Hour; 5 Calendar Days	10 Calendar Days

Hospitalization is defined as initial hospitalization or prolongation of hospitalization for ≥ 24 hours, due to adverse event related to study procedures.

13.4. Submit written adverse event reports in one of the following ways:

(1) PDF by email:	Cathleen_lesniewski@urmc.rochester.edu
(2) By mail:	**Cathleen Lesniewski**<br>URCC NCORP Research Base<br>Saunders Research Building<br>265 Crittenden Blvd<br>CU 420658<br>Rochester, NY 14642
(3) By fax:	Cathleen Lesniewski<br>585-461-5601

13.5. An *unexpected* adverse event is defined as any adverse experience, the specificity or severity of which is not consistent with the risk information described in section 13.1.

13.6. A serious event refers to any event in which the outcome results in any of the following: death, a life-threatening adverse experience, inpatient hospitalization or prolongation of existing hospitalization, a persistent or significant disability, incapacity, or a congenital anomaly/birth defect. Important medical events that may not result in death, be life-threatening, or require hospitalization may be considered a serious adverse drug experience when, based upon appropriate medical judgment, they may jeopardize the participant and may require medical or surgical intervention to prevent one of the outcomes listed in this definition.

13.7. ONLY serious adverse events related to the study procedures need to be reported for data and safety monitoring purposes. AEs and SAEs related to routine oncology treatment and care DO NOT need to be reported, but will be collected on outcomes forms as per section 7.1.

13.8. Adverse events should be reported to the local IRB as per their requirements.

13.9. Data Safety and Monitoring

13.9.1. All adverse events requiring reporting will be submitted to the Research Base as described in section 13.4. Adverse events that are *serious* AND *unexpected* AND *related* will be forwarded to the

study chair and the URCC Data Safety and Monitoring Committee (DSMC) chair immediately upon receipt at URCC. Additional information may be requested upon their review.

13.9.2. All adverse events reported to URCC are entered into a protocol-specific spreadsheet. Adverse event rates are monitored utilizing the spreadsheet. If a serious adverse event is being reported frequently, the study chair will conduct a detailed review. The DSMC Committee Chair will be notified and will determine if further action is required.

13.9.3. The URCC Data Safety Monitoring Committee (DSMC) will review study progress and cumulative reports of adverse events at annual meetings. An overall assessment of accrual and adverse events will enable the committee members to assess whether significant benefits or risks are occurring that would warrant study closure.

13.9.4. The URCC will notify the NCORPs immediately of any serious safety concerns identified by the DSMC. DSMC reports will be available for download on the Research Base website.

14. CRITERIA FOR WITHDRAWAL

If an oncology physician withdraws from study, no further patients with this oncology physician will be recruited. The final physician follow-up survey should be completed at the end of the study or at the time of a physician withdrawing; for example, if they were to move or join another practice. Whenever possible, participants that have already been enrolled should complete study procedures (see section 11.4.1c). The URCC Research NCORP Base should be contacted to discuss follow up procedures for participants whose enrolled physician is withdrawing from the study.

See section 11.4.1c for procedures related to missing data, lost to follow up, or withdrawal. Participants that decline or are too ill to complete follow-up surveys should not be considered as withdrawals; instead they will be considered active participants with missing survey data. Due to the nature of the illness, we expect there will be participants that will be too ill to complete their study related procedures (e.g., surveys, GA procedures). In these situations, CRAs should still be collecting medical record chart review and completing CRA forms as per section 11.4.1c.

Patients can partially or completely discontinue their own participation in study activities at any time without actively (verbally) withdrawing from the study (i.e., active participant with missing data). A withdrawal from the study is defined as a patient who verbally states that they no longer wish to be participating in, contacted or associated with this study. Only a patient can withdraw themselves from the study. If the patient does not have cognitive capacity, their proxy or authorized representative can withdraw a patient from the study. CRAs cannot withdraw a patient from study.

Because of the fragile patient population being studied, the patient should not be automatically assumed to be withdrawn from the study if they are too ill to complete surveys or if they decline to complete surveys or GA procedures at one or more visits or go into hospice. The CRA should continue to obtain medical chart data, as it relates to subject outcomes, treatment and toxicity outcomes (see Section 11.4.1c) for both active with missing data and withdrawal patients unless the patient provides in writing that they no longer wish this data to be collected. This should be provided to the UR NCORP Research Base. These procedures do not require patient participation. Research Base staff will also obtain Medicare data if agreed to in the patient consent.

All data will be kept in a confidential manner. All data will be used unless permission is revoked in writing and forwarded to the URCC NCORP Research Base. Site staff should forward any such correspondence to the URCC NCORP Research Base.

15. STATISTICAL PLAN

15.1. Statistical Considerations

Unless otherwise stated, all statistical tests will be performed at the two-tailed 5% level of significance. Likewise, 95% confidence intervals will be constructed for effect estimates. Data will be analyzed on an "intent-to-treat" basis. The assumptions underlying all statistical analyses will be thoroughly checked using appropriate graphical and numerical methods.[110,111] In case of serious violations of distributional assumptions such as normality, appropriate transformations or nonparametric methods will be performed.[112,113] If outliers or influential data are detected, the accuracy of the data will be investigated. If no errors are found, analyses will be repeated after removing these cases to evaluate their impact on the results. However, the final analyses will include these data points.

This is a cluster-randomized trial with NCORP practice sites being the clusters. The analyses involve use of mixed models that take into consideration possible correlation among the participants within a cluster. This is accomplished by including NCORP site as a random effect in all the models below. Intra-cluster correlation (ICC) will be calculated from the variance component estimates as Var(NCORP) / [Var(NCORP) + Var(Residual)]. To estimate uncertainty in the calculated ICCs, we will use Bayesian methods (Markov Chain Monte-Carlo) assuming a noninformative prior to estimate credible intervals for the computed ICCs. The specific NCORP differences will be assessed graphically using Best Linear Unbiased Predictors (BLUP) of the mean response for each NCORP.

15.2. Justification of Study Design

The study is designed as a cluster randomized trial because a care or service model is applied to each participant by the oncology team. If a cluster design were not undertaken, there would be contamination in that practitioners and teams could choose the care or service model once they were exposed to participants in both arms. The cluster randomized design will allow for the comparison of toxicity between Arms 1 and 2 in the same timeframe.

15.3. Sample Size Considerations

<u>*Cluster Randomized Design Sample Size:*</u> The primary outcome measure for this study is the proportion of participants that experience grade 3-5 toxicity within 3 months of treatment initiation. Given the clinical significance of toxicity, we propose that any statistically significant reduction in the proportion of participants that experience toxicity would be clinically significant. In the published CARG study, 307 of 500 participants had advanced cancer and of these, 141 (46%) experienced grade 3, 4, or 5 toxicity within 3 months.[5] The proportion of participants that underwent full geriatric assessment plus interventions in our geriatric oncology clinic that experienced chemotherapy toxicity was 33%. Our participant population aged 70 and over with advanced solid tumor malignancies receiving chemotherapy, was similar to participants studied in our preliminary work. Our previous multicenter study[5] has allowed us to calculate the intracluster correlation (ICC) amongst 7 different sites for the assessment of the primary outcome, toxicity. The ICC was low, 0.002, which likely reflects the standard way that oncology physicians and their teams assess chemotherapy toxicity with NCI Common Toxicity Criteria. To be on the conservative side, our power calculations assume ICC=0.10. This design (8 sites per arm and 43 participants per site) has 80% power to detect a 13% reduction in the proportion of participants that experience grade 3-5

Table 2: Power Calculations

P0: 0.46

P0-P1	P1	Power	CCOPS/Arm	N/Cluster	N Total
0.10	0.36	0.8	8	69	1104
0.12	0.34	0.8	8	45	720
**0.13**	**0.33**	**0.8**	**8**	**39**	**624**
0.14	0.32	0.8	8	32	512
0.15	0.31	0.8	8	28	448

P0=estimated proportion for usual care; P1=proportion of toxicity in intervention arm; Intracluster correlation=0.10; Power=80%; Two-sided significance level=0.05

chemotherapy toxicity within 3 months of chemotherapy initiation, assuming a two-sided significance level of 0.05 and an ICC of 0.10. See Table 2 for sample size requirements for some other changes in proportion of toxicity. Accounting for a small drop-out rate of 10% (based on our observational cohort data[5]), the targeted accrual will be 700 participants total. Because toxicity is assessed from the medical record and primary team, the drop-out rate reflects participants that sign consent but withdraw prior to baseline assessment.

During NCORP site recruitment, if more than 16 NCORP sites are interested in participating, we will allow randomization. The total participant sample size will remain the same, and accrual will cease when that target is met.

15.4. Primary Analysis

a. The primary outcome measure for this study is the proportion of participants that experience grade 3-5 toxicity within 3 months of treatment initiation. Because of the cluster randomized study design, we will apply generalized linear mixed model (GLMM) methodology.[114] Toxicity (Yes/No) will be the response, and arm, treatment type and arm:treatment type interaction will be the fixed effects. NCORP practice site will be entered as a random effect independent of residual error. Estimation will be performed using the Residual Pseudo Likelihood procedure, assuming a binomial distribution and logit link. Using the fitted model, we will provide estimates and 95% confidence intervals for proportion of participants that experience toxicity for each arm, as well as risk ratios between the arms. This will allow the results for those who fit the original eligibility criterion (on chemotherapy) to be compared to the other allowed treatments that have a similar toxicity profile as chemotherapy.

15.5.Secondary Analyses

15.5.1. Secondary Aim 1. We will determine the effect of the intervention on 6-month survival using log rank tests and survival plots. Assuming an exponential survival distribution, and given survival proportion of 0.89 at 6 months from our previous observational work[5] and a sample size of 624, we estimate that there will be 80% power (0.05 significance level) to detect an increase in survival proportion greater than 0.942, implying a detectable hazard ratio of 0.511.

15.5.2. Secondary Aim 2a. We will compare the effect of the intervention on measures of decisional regret (both participant and oncology physician) using four linear mixed models (regret). For each model, Arm will be the fixed effect and NCORP will be a random effect (independent of residual error). Restricted Maximum Likelihood (REML) estimation will be used, and inference will be performed using the Kenward-Roger degrees of freedom adjustment procedure. We will also determine whether the intervention influences the relative dose intensity (RDI) of treatment given in the first line setting by analyzing the Phase II RDI in the same manner. In addition, to investigate whether the intervention changes dosing, we will analyze with a linear mixed model. In this model, RDI will be the response. The fixed effect will be Arm and the random effect will be NCORP. Overall change in dosing will be assessed with an F Test. Lastly, we will determine the association of baseline oncology physician and patient decision-making interaction on likelihood of developing toxicity. Variables to be evaluated will be derived from participant assessments (control preferences (CP)) and oncology physician comfort (PC) with shared decision-making. A GLMM will be fit with chemotherapy toxicity as the response, Arm, , CP and PC as fixed factors, and NCORP site as a random effect independent of residual error. Otherwise, the modeling methodology is the same as for the Primary Aim.

15.5.3. Secondary Aim 2b. We will compare whether the uptake of geriatric assessment interventions (% of recommended interventions carried out influences chemotherapy toxicity. The data from the intervention arm will be fit to a GLMM with toxicity as the outcome, percent of recommended interventions as the fixed effect, and NCORP site as a random effect independent of residual error. Otherwise, the modeling methodology is the same as for the Primary Aim.

15.5.4. Exploratory Aims. We will evaluate changes in functional abilities and physical performance between Arms 1 and 2. For each of these outcomes, a GLMM structured the same as that used in Specific Aim 2 will be used. Functional status will measured with IADL score. Physical performance will be measured with the OARS Physical Health Subscale and the Short Physical Performance Battery. PRO-CTCAE analyses will be performed using descriptive statistics, correlations, and regressions to evaluate changes in symptoms over time, relationship between PRO results and clinician-related to toxicity, and relationship of symptoms with treatment decisions.

15.6. Missing Data

Every effort will be made to encourage and facilitate participants' completion of questionnaires, but because of dropout, missing data will occur. We will evaluate the patterns of missing data and associations of missingness with other available variables. Under the missing at random (MAR) assumption, we will use multiple imputation to obtain unbiased estimates of the key statistics. If the data are suspected to be missing not at random (MNAR), a sensitivity analysis using selection and/or pattern-mixture models will be run to determine the impact on the results.[115] If the estimates are similar to the ones obtained from the simpler analysis of only complete cases, we will report the complete-case analysis results.

It is hypothesized that the GA summary and GA-driven recommendations provided in the intervention arm will result in a greater number of missing data (e.g., if participants decide not to go onto a new treatment regimen after reviewing the summary) so for these participants, no toxicities will be reported. We will impute this kind of missing data with the data from the PRO-CTCAE. We will also still follow the participants. This will enable us to perform ITT analysis.[116] Missing data for other reason, e.g., dropout, will be handled with the methodologies described in the previous paragraph.

16. DATA MANAGEMENT AND QUALITY ASSURANCE

16.1. Training Procedures

A special training session was held at the annual URCC NCORP Research Base meeting in September 2013. This training included a detailed review of the study rationale, design, and research administration procedures. Training sessions will be held with the staff from each site via teleconference and at the annual meetings. These sessions and the corresponding procedures manuals will review the following: 1) informed consent; 2) completing the assessments using Teleforms; 3) completing the functional and objective measures; 4) data collection via chart extraction; 5) completing the web-based intervention using mycarg.org (for intervention arm only); 6) transfer of the data to the URCC NCORP Research Base; 7) formulating the research chart; and 8) a discussion of interviewing techniques so that the research team will standardize their approaches in order to elicit consistent data from participants. There will be a protocol update every year at the annual Research Base meeting. All assessments, data collection forms, and manuals will be readily available on the NCORP Research Base website.

16.2. Data Management

The same protocols and procedures for data quality and control that we use for all URCC NCORP Research Base protocols (which accrued over 1,000 participants in the previous year) will be used for this study. Once the patient consents to the protocol, he/she will be assigned a unique identifier by the Research Base, which will be used to link all participant data. Oncology physician and participant assessments will be captured using scannable Teleforms. The CRA and/or Research Nurse at each site

will ensure that data are complete prior to submission. Assessment of cancer characteristics, cancer treatment and RDI, and toxicity outcomes will be captured via Teleforms. At the Research Base, data are scanned into an electronic password-secured Access database which is backed up every 24 hours. At the Research Base, staff will ensure that all data are collected in order to minimize missing data by employing multi-step verification procedures and querying originating sites for missing or ambiguous data. Queries will be reviewed with the sites, especially regarding the ascertainment of toxicity.

16.3. REDCap

Data are also collected and managed by the research teams at University of Rochester Medical Center using REDCap electronic data capture tools hosted at URMC. We will evaluate records, clinical characteristics, and outcomes and we will utilize REDCap to collect and manage this information. Further, we will link this information to the encrypted ACCESS database (which contains the survey information) with a unique identifier.

16.3.1. The University of Rochester Medical Center provides the following information on the **REDCap program: "**Vanderbilt University, in collaboration with a consortium of institutional partners, has developed a software toolset and workflow methodology for electronic collection and management of research and clinical trial data, called REDCap (Research Electronic Data Capture). The REDCap system is a secure, web-based application that is flexible enough to be used for a variety of types of research. It provides an intuitive interface for users to enter data and real time validation rules (with automated data type and range checks) at the time of data entry. REDCap offers easy data manipulation with audit trails and functionality for reporting, monitoring and querying participant records, as well as an automated export mechanism to common statistical packages (SPSS, SAS, Stata, R/S-Plus). Through the REDCap Consortium, Vanderbilt has disseminated REDCap for use around the world. Currently, over 240 academic and non-profit consortium partners on six continents with over 26,000 research end-users use REDCap."

16.3.2. According to the University of Rochester Clinical and Translational Science Institute (CTSI), REDCap is supported with the following means. "The *CTSI Informatics Core*, a unit of the SMD *Academic Information Technology (AIT) Group,* will serve as a central facilitator for data processing and management. REDCap data collection projects rely on a thorough study-specific data dictionary defined in an iterative self-documenting process by all members of the research team, with planning assistance from the *AIT-CTSI Informatics Core*. The iterative development and testing process results in a well-planned data collection strategy for individual studies."

16.3.3. The CTSI states that regarding security, "REDCap servers are housed in a local data center at the University of Rochester and all web-based information transmission is encrypted. REDCap was developed in a manner consistent with HIPAA security requirements and is recommended to University of Rochester researchers by the URMC Research Privacy Officer and Office for Human Subject Protection.

16.4. Data Storage

All written materials will be kept confidential, locked in the private offices and limited-access file room of the URCC NCORP Research Base and identified by ID numbers. All electronic information will be kept confidential with password-protected, limited access.

The Case Summary should accompany ALL data submissions. All completed forms must be submitted should be sent within 7 days of study visit to:

Libby Nagalski
URCC NCORP Research Base
Saunders Research Building

265 Crittenden Blvd
Box CU 420658
Rochester, NY 14642

17. DATA COLLECTION AND MEASURES

For a detailed description of the measures that will be collected, refer to Appendix IIA-IID: Summary of Measures, Participant Measures, Clinical Research Associate Materials, and Oncology physician Measures.

18. PARTICIPANT CONSENT AND PEER JUDGMENT

All investigational, FDA, NCI, state, federal and institutional regulations concerning informed consent and peer judgment will be fulfilled.

19. RECORD AND DATA RETENTION

Clinical research records are source documents and records, in any form (including, but not limited to, written, electronic, magnetic, and optical records, in addition, scans (x-rays and electrocardiograms) that describe or record the methods, conduct, and/or results of a trial, and the actions taken. Unlike pharmaceutical-sponsored research, under the Terms of the NIH Award, the awardee institution retains ownership of the clinical research records that were conducted with NIH support. Records may be preserved in hardcopy, electronic or other media form since there is no regulatory requirement that clinical research records be retained in a certain type of format. However, investigators should check with their institution for institutional policies and procedures pertaining to record retention. All records relating to research that is conducted must be retained for at least five years after completion of the research. The three-year time period begins when the individual institution's engagement in the human subject's research activity ends. Human subject research activities are considered completed once all research-related interventions and interactions with human subjects have been completed, all data collection and analysis of identifiable private information described in the IRB-approved research plan have been finished and primary analysis of either identifiable private or de-identified information is completed.

20. REFERENCES

1. Hutchins LF, Unger JM, Crowley JJ, Coltman CA, Jr., Albain KS. Underrepresentation of patients 65 years of age or older in cancer-treatment trials. *The New England journal of medicine.* 1999;341(27):2061-2067.
2. Murthy VH, Krumholz HM, Gross CP. Participation in cancer clinical trials: race-, sex-, and age-based disparities. *JAMA : the journal of the American Medical Association.* 2004;291(22):2720-2726.
3. Dale W, Mohile SG, Eldadah BA, et al. Biological, clinical, and psychosocial correlates at the interface of cancer and aging research. *Journal of the National Cancer Institute.* 2012;104(8):581-589.
4. Hurria A, Mohile SG, Dale W. Research priorities in geriatric oncology: addressing the needs of an aging population. *Journal of the National Comprehensive Cancer Network : JNCCN.* 2012;10(2):286-288.
5. Hurria A, Togawa K, Mohile SG, et al. Predicting chemotherapy toxicity in older adults with cancer: a prospective multicenter study. *Journal of clinical oncology : official journal of the American Society of Clinical Oncology.* 2011;29(25):3457-3465.
6. Indurkhya A, Mitra N, Schrag D. Using propensity scores to estimate the cost-effectiveness of medical therapies. *Statistics in medicine.* 2006;25(9):1561-1576.
7. Yabroff KR, Lamont EB, Mariotto A, et al. Cost of care for elderly cancer patients in the United States. *Journal of the National Cancer Institute.* 2008;100(9):630-641.
8. Schnipper LE, Smith TJ, Raghavan D, et al. American Society of Clinical Oncology identifies five key opportunities to improve care and reduce costs: the top five list for oncology. *Journal of clinical oncology : official journal of the American Society of Clinical Oncology.* 2012;30(14):1715-1724.
9. Extermann M, Aapro M, Bernabei R, et al. Use of comprehensive geriatric assessment in older cancer patients: recommendations from the task force on CGA of the International Society of Geriatric Oncology (SIOG). *Critical reviews in oncology/hematology.* 2005;55(3):241-252.
10. Balducci L, Cohen HJ, Engstrom PF, et al. Senior adult oncology clinical practice guidelines in oncology. *Journal of the National Comprehensive Cancer Network : JNCCN.* 2005;3(4):572-590.
11. Balducci L, Extermann M. Management of cancer in the older person: a practical approach. *The oncologist.* 2000;5(3):224-237.
12. Hurria A, Gupta S, Zauderer M, et al. Developing a cancer-specific geriatric assessment: a feasibility study. *Cancer.* 2005;104(9):1998-2005.
13. Hurria A, Browner IS, Cohen HJ, et al. Senior adult oncology. *Journal of the National Comprehensive Cancer Network : JNCCN.* 2012;10(2):162-209.
14. Pal SK, Katheria V, Hurria A. Evaluating the older patient with cancer: understanding frailty and the geriatric assessment. *CA: a cancer journal for clinicians.* 2010;60(2):120-132.
15. Rodin MB, Mohile SG. A practical approach to geriatric assessment in oncology. *Journal of clinical oncology : official journal of the American Society of Clinical Oncology.* 2007;25(14):1936-1944.
16. Extermann M, Hurria A. Comprehensive geriatric assessment for older patients with cancer. *Journal of clinical oncology : official journal of the American Society of Clinical Oncology.* 2007;25(14):1824-1831.
17. Stuck AE, Siu AL, Wieland GD, Adams J, Rubenstein LZ. Comprehensive geriatric assessment: a meta-analysis of controlled trials. *Lancet.* 1993;342(8878):1032-1036.
18. Stuck AE, Aronow HU, Steiner A, et al. A trial of annual in-home comprehensive geriatric assessments for elderly people living in the community. *The New England journal of medicine.* 1995;333(18):1184-1189.
19. Cohen HJ, Feussner JR, Weinberger M, et al. A controlled trial of inpatient and outpatient geriatric evaluation and management. *The New England journal of medicine.* 2002;346(12):905-912.

20. Hurria A, Lachs MS, Cohen HJ, Muss HB, Kornblith AB. Geriatric assessment for oncologists: rationale and future directions. *Critical reviews in oncology/hematology.* 2006;59(3):211-217.
21. Ingram SS, Seo PH, Martell RE, et al. Comprehensive assessment of the elderly cancer patient: the feasibility of self-report methodology. *Journal of clinical oncology : official journal of the American Society of Clinical Oncology.* 2002;20(3):770-775.
22. Huerta MF, Farber GK, Wilder EL, et al. NIH Roadmap interdisciplinary research initiatives. *PLoS computational biology.* 2005;1(6):e59.
23. Hurria A, Cirrincione CT, Muss HB, et al. Implementing a geriatric assessment in cooperative group clinical cancer trials: CALGB 360401. *Journal of clinical oncology : official journal of the American Society of Clinical Oncology.* 2011;29(10):1290-1296.
24. Mohile SG, Xian Y, Dale W, et al. Association of a cancer diagnosis with vulnerability and frailty in older Medicare beneficiaries. *Journal of the National Cancer Institute.* 2009;101(17):1206-1215.
25. Mohile SG, Fan L, Reeve E, et al. Association of cancer with geriatric syndromes in older Medicare beneficiaries. *Journal of clinical oncology : official journal of the American Society of Clinical Oncology.* 2011;29(11):1458-1464.
26. Jemal A, Siegel R, Xu J, Ward E. Cancer statistics, 2010. *CA Cancer J Clin.*60(5):277-300.
27. Smith BD, Smith GL, Hurria A, Hortobagyi GN, Buchholz TA. Future of Cancer Incidence in the United States: Burdens Upon an Aging, Changing Nation. *Journal of Clinical Oncology.* 2009;27(17):2758-2765.
28. Mohile S, Dale W, Hurria A. Geriatric oncology research to improve clinical care. *Nature reviews. Clinical oncology.* 2012.
29. Mohile S, Dale W, Hurria A. Geriatric oncology research to improve clinical care. *Nature reviews. Clinical oncology.* 2012;9(10):571-578.
30. Crome P, Flanagan RJ. Pharmacokinetic studies in elderly people. Are they necessary? *Clin Pharmacokinet.* 1994;26(4):243-247.
31. Koroukian SM, Murray P, Madigan E. Comorbidity, disability, and geriatric syndromes in elderly cancer patients receiving home health care. *Journal of clinical oncology : official journal of the American Society of Clinical Oncology.* 2006;24(15):2304-2310.
32. Repetto L. Greater risks of chemotherapy toxicity in elderly patients with cancer. *The journal of supportive oncology.* 2003;1(4 Suppl 2):18-24.
33. Jensen SA, Vilmar A, Sorensen JB. Adjuvant chemotherapy in elderly patients (>or=75 yr) completely resected for colon cancer stage III compared to younger patients: toxicity and prognosis. *Medical oncology (Northwood, London, England).* 2006;23(4):521-531.
34. Garg P, Rana F, Gupta R, Buzaianu EM, Guthrie TH. Predictors of toxicity and toxicity profile of adjuvant chemotherapy in elderly breast cancer patients. *The breast journal.* 2009;15(4):404-408.
35. Katz S, Ford AB, Moskowitz RW, Jackson BA, Jaffe MW. Studies of illness in the aged, the index of ADL: A standardized measure of biological and psychological function. *JAMA : the journal of the American Medical Association.* 1963;185:914-919.
36. Lawton MP. Scales to measure competence in everyday activities. *Psychopharmacology bulletin.* 1988;24(4):609-614.
37. Guralnik JM, Ferrucci L, Simonsick EM, Salive ME, Wallace RB. Lower-extremity function in persons over the age of 70 years as a predictor of subsequent disability. *The New England journal of medicine.* 1995;332(9):556-561.
38. Charlson ME, Pompei P, Ales KL, MacKenzie CR. A new method of classifying prognostic comorbidity in longitudinal studies: development and validation. *J Chronic Dis.* 1987;40(5):373-383.
39. Ware JE, Jr., Sherbourne CD. The MOS 36-item short-form health survey (SF-36). I. Conceptual framework and item selection. *Medical care.* 1992;30(6):473-483.
40. Pfeiffer E. A short portable mental status questionnaire for the assessment of organic brain deficit in elderly patients. *Journal of the American Geriatrics Society.* 1975;23(10):433-441.

41. Juurlink DN, Mamdani M, Kopp A, Laupacis A, Redelmeier DA. Drug-drug interactions among elderly patients hospitalized for drug toxicity. *JAMA : the journal of the American Medical Association.* 2003;289(13):1652-1658.
42. Fillenbaum G, Heyman A, Williams K, Prosnitz B, Burchett B. Sensitivity and specificity of standardized screens of cognitive impairment and dementia among elderly black and white community residents. *Journal of clinical epidemiology.* 1990;43(7):651-660.
43. Guralnik JM, Simonsick EM, Ferrucci L, et al. A short physical performance battery assessing lower extremity function: association with self-reported disability and prediction of mortality and nursing home admission. *Journal of gerontology.* 1994;49(2):M85-94.
44. Hays RD, Sherbourne CD, Mazel RM. The RAND 36-Item Health Survey 1.0. *Health economics.* 1993;2(3):217-227.
45. Podsiadlo D, Richardson S. The timed "Up & Go": a test of basic functional mobility for frail elderly persons. *Journal of the American Geriatrics Society.* 1991;39(2):142-148.
46. Stewart AL, Kamberg CJ. In: Stewart AL, Ware JE, Jr., eds. *Measuring functioning and well-being: The Medical Outcomes Study approach.* Durham, North Carolina: Duke University Press; 1992:86-101.
47. Fillenbaum GG, Smyer MA. The development, validity, and reliability of the OARS multidimensional functional assessment questionnaire. *Journal of gerontology.* 1981;36(4):428-434.
48. George LK, Fillenbaum GG. OARS methodology. A decade of experience in geriatric assessment. *Journal of the American Geriatrics Society.* 1985;33(9):607-615.
49. Kawas C, Karagiozis H, Resau L, Corrada M, Brookmeyer R. Reliability of the Blessed Telephone Information-Memory-Concentration Test. *Journal of geriatric psychiatry and neurology.* 1995;8(4):238-242.
50. Nasreddine ZS, Phillips NA, Bedirian V, et al. The Montreal Cognitive Assessment, MoCA: a brief screening tool for mild cognitive impairment. *Journal of the American Geriatrics Society.* 2005;53(4):695-699.
51. Fried LP, Ferrucci L, Darer J, Williamson JD, Anderson G. Untangling the concepts of disability, frailty, and comorbidity: implications for improved targeting and care. *The journals of gerontology. Series A, Biological sciences and medical sciences.* 2004;59(3):255-263.
52. Bandeen-Roche K, Xue QL, Ferrucci L, et al. Phenotype of frailty: characterization in the women's health and aging studies. *The journals of gerontology. Series A, Biological sciences and medical sciences.* 2006;61(3):262-266.
53. Bylow K, Hemmerich J, Mohile SG, Stadler WM, Sajid S, Dale W. Obese frailty, physical performance deficits, and falls in older men with biochemical recurrence of prostate cancer on androgen deprivation therapy: a case-control study. *Urology.* 2011;77(4):934-940.
54. Klepin H, Mohile S, Hurria A. Geriatric assessment in older patients with breast cancer. *Journal of the National Comprehensive Cancer Network : JNCCN.* 2009;7(2):226-236.
55. Mohile SG, Bylow K, Dale W, et al. A pilot study of the vulnerable elders survey-13 compared with the comprehensive geriatric assessment for identifying disability in older patients with prostate cancer who receive androgen ablation. *Cancer.* 2007;109(4):802-810.
56. Rao AV, Hsieh F, Feussner JR, Cohen HJ. Geriatric evaluation and management units in the care of the frail elderly cancer patient. *The journals of gerontology. Series A, Biological sciences and medical sciences.* 2005;60(6):798-803.
57. Karnofsky DA, Ellison RR, Golbey RB. Selection of patients for evaluation of chemotherapeutic procedures in advanced cancer. *J Chronic Dis.* 1962;15:243-249.
58. Zubrod C, Schniderman M, Frei E. Appraisal of methods for the study of chemotherapy of cancer in man. *Journal of Chronic Diseases.* 1960(11):7-33.
59. Albain KS, Crowley JJ, Hutchins L, et al. Predictors of survival following relapse or progression of small cell lung cancer. Southwest Oncology Group Study 8605 report and analysis of recurrent disease data base. *Cancer.* 1993;72(4):1184-1191.

60. Albain KS, Crowley JJ, LeBlanc M, Livingston RB. Survival determinants in extensive-stage non-small-cell lung cancer: the Southwest Oncology Group experience. *Journal of clinical oncology : official journal of the American Society of Clinical Oncology.* 1991;9(9):1618-1626.
61. Hurria A. We need a geriatric assessment for oncologists. *Nature clinical practice. Oncology.* 2006;3(12):642-643.
62. Hurria A. Incorporation of geriatric principles in oncology clinical trials. *Journal of clinical oncology : official journal of the American Society of Clinical Oncology.* 2007;25(34):5350-5351.
63. Keating NL, Norredam M, Landrum MB, Huskamp HA, Meara E. Physical and mental health status of older long-term cancer survivors. *Journal of the American Geriatrics Society.* 2005;53(12):2145-2152.
64. Maione P, Perrone F, Gallo C, et al. Pretreatment quality of life and functional status assessment significantly predict survival of elderly patients with advanced non-small-cell lung cancer receiving chemotherapy: a prognostic analysis of the multicenter Italian lung cancer in the elderly study. *Journal of clinical oncology : official journal of the American Society of Clinical Oncology.* 2005;23(28):6865-6872.
65. Stafford RS, Cyr PL. The impact of cancer on the physical function of the elderly and their utilization of health care. *Cancer.* 1997;80(10):1973-1980.
66. Audisio RA, Gennari R, Sunouchi K, et al. Preoperative assessment of cancer in elderly patients: a pilot study. *Supportive cancer therapy.* 2003;1(1):55-60.
67. Stone CA, Lawlor PG, Savva GM, Bennett K, Kenny RA. Prospective study of falls and risk factors for falls in adults with advanced cancer. *Journal of clinical oncology : official journal of the American Society of Clinical Oncology.* 2012;30(17):2128-2133.
68. Extermann M, Balducci L, Lyman GH. What threshold for adjuvant therapy in older breast cancer patients? *Journal of clinical oncology : official journal of the American Society of Clinical Oncology.* 2000;18(8):1709-1717.
69. Frasci G, Lorusso V, Panza N, et al. Gemcitabine plus vinorelbine versus vinorelbine alone in elderly patients with advanced non-small-cell lung cancer. *Journal of clinical oncology : official journal of the American Society of Clinical Oncology.* 2000;18(13):2529-2536.
70. Firat S, Byhardt RW, Gore E. Comorbidity and Karnofksy performance score are independent prognostic factors in stage III non-small-cell lung cancer: an institutional analysis of patients treated on four RTOG studies. Radiation Therapy Oncology Group. *International journal of radiation oncology, biology, physics.* 2002;54(2):357-364.
71. Piccirillo JF, Tierney RM, Costas I, Grove L, Spitznagel EL, Jr. Prognostic importance of comorbidity in a hospital-based cancer registry. *JAMA : the journal of the American Medical Association.* 2004;291(20):2441-2447.
72. Satariano WA, Ragland DR. The effect of comorbidity on 3-year survival of women with primary breast cancer. *Annals of internal medicine.* 1994;120(2):104-110.
73. Gronberg BH, Sundstrom S, Kaasa S, et al. Influence of comorbidity on survival, toxicity and health-related quality of life in patients with advanced non-small-cell lung cancer receiving platinum-doublet chemotherapy. *Eur J Cancer.* 2010;46(12):2225-2234.
74. Jung SY, Rosenzweig M, Linkov F, Brufsky A, Weissfeld JL, Sereika SM. Comorbidity as a Mediator of Survival Disparity Between Younger and Older Women Diagnosed With Metastatic Breast Cancer. *Hypertension.* 2011.
75. Albertsen PC, Moore DF, Shih W, Lin Y, Li H, Lu-Yao GL. Impact of comorbidity on survival among men with localized prostate cancer. *Journal of clinical oncology : official journal of the American Society of Clinical Oncology.* 2011;29(10):1335-1341.
76. Steyerberg EW, Neville BA, Koppert LB, et al. Surgical mortality in patients with esophageal cancer: development and validation of a simple risk score. *Journal of clinical oncology : official journal of the American Society of Clinical Oncology.* 2006;24(26):4277-4284.
77. Maggiore RJ, Gross CP, Hurria A. Polypharmacy in older adults with cancer. *The oncologist.* 2010;15(5):507-522.

78. Landi F, Zuccala G, Gambassi G, et al. Body mass index and mortality among older people living in the community. *Journal of the American Geriatrics Society.* 1999;47(9):1072-1076.
79. Dewys WD, Begg C, Lavin PT, et al. Prognostic effect of weight loss prior to chemotherapy in cancer patients. Eastern Cooperative Oncology Group. *The American journal of medicine.* 1980;69(4):491-497.
80. Wadley VG, Okonkwo O, Crowe M, Ross-Meadows LA. Mild cognitive impairment and everyday function: evidence of reduced speed in performing instrumental activities of daily living. *The American journal of geriatric psychiatry : official journal of the American Association for Geriatric Psychiatry.* 2008;16(5):416-424.
81. Sauvaget C, Yamada M, Fujiwara S, Sasaki H, Mimori Y. Dementia as a predictor of functional disability: a four-year follow-up study. *Gerontology.* 2002;48(4):226-233.
82. Dodge HH, Kadowaki T, Hayakawa T, Yamakawa M, Sekikawa A, Ueshima H. Cognitive impairment as a strong predictor of incident disability in specific ADL-IADL tasks among community-dwelling elders: the Azuchi Study. *The Gerontologist.* 2005;45(2):222-230.
83. Hurria A, Li D, Hansen K, et al. Distress in older patients with cancer. *Journal of clinical oncology : official journal of the American Society of Clinical Oncology.* 2009;27(26):4346-4351.
84. Seeman TE, Berkman LF, Kohout F, Lacroix A, Glynn R, Blazer D. Intercommunity variations in the association between social ties and mortality in the elderly. A comparative analysis of three communities. *Annals of epidemiology.* 1993;3(4):325-335.
85. Kroenke CH, Kubzansky LD, Schernhammer ES, Holmes MD, Kawachi I. Social networks, social support, and survival after breast cancer diagnosis. *Journal of clinical oncology : official journal of the American Society of Clinical Oncology.* 2006;24(7):1105-1111.
86. Kornblith AB, Herndon JE, 2nd, Zuckerman E, et al. Social support as a buffer to the psychological impact of stressful life events in women with breast cancer. *Cancer.* 2001;91(2):443-454.
87. Hurria A, Cohen HJ, Extermann M. Geriatric Oncology Research in the Cooperative Groups: A Report of a SIOG Special Meeting. *Journal of geriatric oncology.* 2010;1(1):40-44.
88. Hurria A, Balducci L, Naeim A, et al. Mentoring junior faculty in geriatric oncology: report from the Cancer and Aging Research Group. *Journal of clinical oncology : official journal of the American Society of Clinical Oncology.* 2008;26(19):3125-3127.
89. Caillet P, Canoui-Poitrine F, Vouriot J, et al. Comprehensive geriatric assessment in the decision-making process in elderly patients with cancer: ELCAPA study. *Journal of clinical oncology : official journal of the American Society of Clinical Oncology.* 2011;29(27):3636-3642.
90. Horgan AM, Leighl NB, Coate L, et al. Impact and Feasibility of a Comprehensive Geriatric Assessment in the Oncology Setting: A Pilot Study. *Am J Clin Oncol.* 2011.
91. McCorkle R, Strumpf NE, Nuamah IF, et al. A specialized home care intervention improves survival among older post-surgical cancer patients. *Journal of the American Geriatrics Society.* 2000;48(12):1707-1713.
92. Goodwin JS, Satish S, Anderson ET, Nattinger AB, Freeman JL. Effect of nurse case management on the treatment of older women with breast cancer. *Journal of the American Geriatrics Society.* 2003;51(9):1252-1259.
93. Seymour MT, Thompson LC, Wasan HS, et al. Chemotherapy options in elderly and frail patients with metastatic colorectal cancer (MRC FOCUS2): an open-label, randomised factorial trial. *Lancet.* 2011;377(9779):1749-1759.
94. Andreozzi J, Dale W, Fan L, Mohile SG. Feasibility of Incorporting Geriatric Assessment into Oncology Practice in Real-Time. *American Geriatrics Society National Meeting.* 2011(D104).
95. Guigoz Y. The Mini Nutritional Assessment (MNA) review of the literature--What does it tell us? *The journal of nutrition, health & aging.* 2006;10(6):466-485; discussion 485-467.
96. Yesavage JA, Brink TL, Rose TL, et al. Development and validation of a geriatric depression screening scale: a preliminary report. *Journal of psychiatric research.* 1982;17(1):37-49.

97. Katzman R, Brown T, Fuld P, Peck A, Schechter R, Schimmel H. Validation of a short Orientation-Memory-Concentration Test of cognitive impairment. *The American journal of psychiatry.* 1983;140(6):734-739.
98. Epstein RM, Alper BS, Quill TE. Communicating evidence for participatory decision making. *JAMA : the journal of the American Medical Association.* 2004;291(19):2359-2366.
99. Epstein RM, Street RL, Jr. Shared mind: communication, decision making, and autonomy in serious illness. *Annals of family medicine.* 2011;9(5):454-461.
100. Young HN, Bell RA, Epstein RM, Feldman MD, Kravitz RL. Physicians' shared decision-making behaviors in depression care. *Archives of internal medicine.* 2008;168(13):1404-1408.
101. Arora NK, Street RL, Jr., Epstein RM, Butow PN. Facilitating patient-centered cancer communication: a road map. *Patient education and counseling.* 2009;77(3):319-321.
102. Shields CG, Coker CJ, Poulsen SS, et al. Patient-centered communication and prognosis discussions with cancer patients. *Patient education and counseling.* 2009;77(3):437-442.
103. Dale W, Hemmerich J, Bylow K, Mohile S, Mullaney M, Stadler WM. Patient anxiety about prostate cancer independently predicts early initiation of androgen deprivation therapy for biochemical cancer recurrence in older men: a prospective cohort study. *Journal of clinical oncology : official journal of the American Society of Clinical Oncology.* 2009;27(10):1557-1563.
104. Detmar SB, Muller MJ, Schornagel JH, Wever LD, Aaronson NK. Health-related quality-of-life assessments and patient-physician communication: a randomized controlled trial. *JAMA : the journal of the American Medical Association.* 2002;288(23):3027-3034.
105. Lyman GH. Impact of chemotherapy dose intensity on cancer patient outcomes. *Journal of the National Comprehensive Cancer Network : JNCCN.* 2009;7(1):99-108.
106. Temel JS, Jackson VA, Billings JA, et al. Phase II study: integrated palliative care in newly diagnosed advanced non-small-cell lung cancer patients. *Journal of clinical oncology : official journal of the American Society of Clinical Oncology.* 2007;25(17):2377-2382.
107. Temel J. Complexities of quality of life analysis in non-small cell lung cancer. *The journal of supportive oncology.* 2007;5(1):30-31.
108. Fromme EK, Eilers KM, Mori M, Hsieh YC, Beer TM. How accurate is clinician reporting of chemotherapy adverse effects? A comparison with patient-reported symptoms from the Quality-of-Life Questionnaire C30. *Journal of clinical oncology : official journal of the American Society of Clinical Oncology.* 2004;22(17):3485-3490.
109. Trotti A, Colevas AD, Setser A, Basch E. Patient-reported outcomes and the evolution of adverse event reporting in oncology. *Journal of clinical oncology : official journal of the American Society of Clinical Oncology.* 2007;25(32):5121-5127.
110. Atkinson A, ed *Plots, Transformation, and Regression.* Oxford: Oxford University Press; 1985.
111. Cook R, Weisberg S, eds. London: Chapman and Hall; 1982. Residuals and influence in Regression.
112. Conover W, Iman R. Rank transformations as a bridge between parametric and nonparametric statistics. *American Statistics.* 1981;35:124-133.
113. Conover W, Iman R. On some alternative procedures using ranks for the analysis of experimental designs. *Comm Statist A.* 1976;5:1349-1368.
114. Brown H, Prescott R. *Applied Mixed Models in Medicine.* Wiley; 2006.
115. Little RJA, Rubin DB. *Statistical analysis with missing data.* 2nd ed. New York: John Wiley; 2002.
116. *The Prevention and Treatment of Missing Data in Clinical Trials.* The National Academies Press; 2010.
117. Kyriakou F, Kountourakis P, Papamichael D. Targeted agents: review of toxicity in the elderly metastatic colorectal cancer patients. Target Oncol. 2011; 6(4): 245-251.
118. Meoni G, Cecere F L, Lucherini E, Di Costanzo F. Medical treatment of advanced non-small cell lung cancer in elderly patients: a review of the role of chemotherapy and targeted agents. Journal of Geriatric Oncology. 2013; 4(3): 282-290.

119. Zustovich F, Novara G. Advanced kidney cancer: treating the elderly. Expert Rev Anticancer Ther. 2013; 13(12): 1389-1398.
120. Grothey A, Van Cutsem E, Sobrero A, et al. Regorafenib monotherapy for previously treated metastatic colorectal cancer (CORRECT): an international, multicentre, randomised, placebo-controlled, phase 3 trial. Lancet. 2013; 381(9863): 303-312.
