# Supplementary material for: An Unsupervised Machine Learning Approach to Evaluating the Association of Symptom Clusters With Adverse Outcomes Among Older Adults With Advanced Cancer: A Secondary Analysis of a Randomized Clinical Trial
Source: JAMA Netw Open. 2023 Mar 22;6(3):e234198. doi: 10.1001/jamanetworkopen.2023.4198 (PMC10034574; doi:10.1001/jamanetworkopen.2023.4198)
Supplement: Supplement 2. — eFigure. Study Flow Diagram [file jamanetwopen-e234198-s002.pdf]

Supplemental Online Content

Xu H, Mohamed M, Flannery M, et al. An unsupervised machine learning approach to evaluating the association of symptom clusters with adverse outcomes among older adults with advanced cancer: a secondary analysis of a randomized clinical trial. *JAMA Netw Open*. 2023;6(3):e234198. doi:10.1001/jamanetworkopen.2023.4198

eFigure. Study Flow Diagram

This supplemental material has been provided by the authors to give readers additional information about their work.

© 2023 Xu H et al. *JAMA Network Open.*

eFigure. Study Flow Diagram

Three-month outcomes for patients who died or lost follow up were included using an intent-to-treat approach.

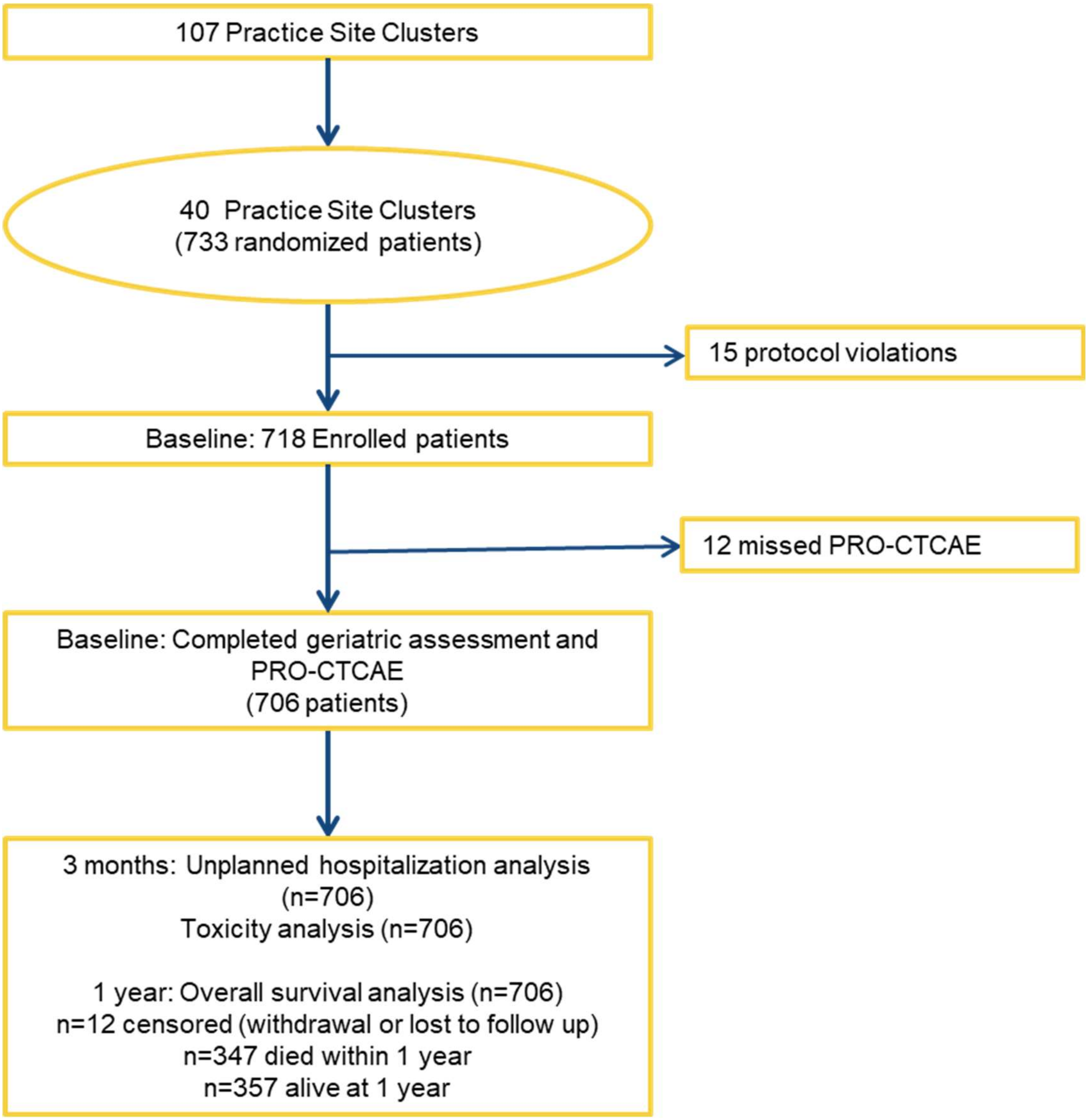

© 2023 Xu H et al. *JAMA Network Open.*
