# Supplementary material for: An Unsupervised Machine Learning Approach to Evaluating the Association of Symptom Clusters With Adverse Outcomes Among Older Adults With Advanced Cancer: A Secondary Analysis of a Randomized Clinical Trial
Source: JAMA Netw Open. 2023 Mar 22;6(3):e234198. doi: 10.1001/jamanetworkopen.2023.4198 (PMC10034574; doi:10.1001/jamanetworkopen.2023.4198)
Supplement: Supplement 3. — Data Sharing Statement [file jamanetwopen-e234198-s003.pdf]

# Data Sharing Statement

Xu. An Unsupervised Machine Learning Approach to Evaluating the Association of Symptom Clusters With Adverse Outcomes Among Older Adults With Advanced Cancer. *JAMA Netw Open*. Published March 22, 2023. doi:10.1001/jamanetworkopen.2023.4198

**Data**
**Data available:** No
